# Efficient Selection of Enhancers and Promoters from MIA PaCa-2 Pancreatic Cancer Cells by ChIP-lentiMPRA

**DOI:** 10.3390/ijms232315011

**Published:** 2022-11-30

**Authors:** Kirill Nikitich Kashkin, Elena Sergeevna Kotova, Irina Vasilievna Alekseenko, Svetlana Sergeevna Bulanenkova, Sergey Borisovich Akopov, Eugene Pavlovich Kopantzev, Lev Grigorievich Nikolaev, Igor Pavlovich Chernov, Dmitry Alexandrovich Didych

**Affiliations:** 1 Shemyakin-Ovchinnikov Institute of Bioorganic Chemistry of the Russian Academy of Sciences, Miklukho-Maklaya, 16/10, 117997 Moscow, Russia; 2 Laboratory of Human Molecular Genetics, Federal Research and Clinical Center of Physical-Chemical Medicine of Federal Medical Biological Agency, Malaya Pirogovskaya Street, 1a, 119435 Moscow, Russia

**Keywords:** ChIP, lentiMPRA, enhancer, promoter, library, pancreatic cancer

## Abstract

A library of active genome regulatory elements (putative promoters and enhancers) from MIA PaCa-2 pancreatic adenocarcinoma cells was constructed using a specially designed lentiviral vector and a massive parallel reporter assay (ChIP-lentiMPRA). Chromatin immunoprecipitation of the cell genomic DNA by H3K27ac antibodies was used for primary enrichment of the library for regulatory elements. Totally, 11,264 unique genome regions, many of which are capable of enhancing the expression of the CopGFP reporter gene from the minimal CMV promoter, were identified. The regions tend to be located near promoters. Based on the proximity assay, we found an enrichment of highly expressed genes among those associated with three or more mapped distal regions (2 kb distant from the 5′-ends of genes). It was shown significant enrichment of genes related to carcinogenesis or Mia PaCa-2 cell identity genes in this group. In contrast, genes associated with 1–2 distal regions or only with proximal regions (within 2 kbp of the 5′-ends of genes) are more often related to housekeeping functions. Thus, ChIP-lentiMPRA is a useful strategy for creating libraries of regulatory elements for the study of tumor-specific gene transcription.

## 1. Introduction

Promoters and enhancers are among the most important elements of the genome that ensure gene transcription [1,2]. In recent years, epigenomic and transcriptomic studies have identified many common structural and functional properties of enhancers and promoters (reviewed in [3,4,5]), which may be related to their evolutionary kinship [6,7]. In the active state, enhancers and promoters are nucleosome-free DNA regions of up to several hundred base pairs occupied by multi-subunit protein complexes.

Both promoters and enhancers can serve as RNA synthesis start sites (for enhancer RNA, or eRNA), often in a bidirectional manner [8,9,10,11,12]. In some cases, enhancers located within genes (intragenic enhancers) can function as alternative tissue-specific promoters, initiating the synthesis of long multiexon polyadenylated transcripts [13,14]. In turn, the promoters of a large proportion of protein-coding and long non-coding RNA genes possess enhancer-like activity, increasing the level of neighboring genes’ transcription [5,15,16,17].

Active enhancers and promoters are typically flanked by nucleosomes that have similar post-translational modifications of histones, such as H3K27Ac (histone H3 acetylated at lysine 27), H3K9Ac, H3K4Me1, or some others [18,19,20,21]. Analysis of the distribution of specific histone marks in the genome is effectively used to predict the localization of enhancers and promoters, as well as to determine their functional state (active, inactive, poised, etc.) [22], reviewed in [23,24]).

In recent years, a number of functional approaches, including MPRA (massively parallel reporter assays), have been developed to identify sequences with enhancer and/or promoter activity in complex mixtures of DNA fragments (DNA libraries) [25,26,27,28,29,30]. The proposed approaches are based on the classical “reporter gene” assay, which consists of the ability of enhancers/promoters to enhance the expression of the reporter gene as part of a particular construct in the cells under study. A fluorescent protein gene is often used as a reporter, which makes it possible to select fluorescent cells using cell sorting (FACS) and obtain an output library enriched with promoter and/or enhancer DNA sequences. Input DNA for MPRA may be produced by DNA synthesis, amplification, genome fragmentation, DNA capture methods [27], or by immunoprecipitation (this work).

The use of lentiviral vectors in the creation of libraries for MPRA [31] makes it possible to obtain cells stably transfected by the studied constructs. Due to the integration of the vectors to the genome, lentiMPRA produces more reproducible results, which correlate with ENCODE annotations and sequence-based models better than episomal assays [32]. Lentiviruses provide an in-genome read-out of the candidate regulatory sequences, can infect many different cells and tissues, even those that may hardly be transfected by episomal vectors, and allow to functionally test thousands of prospective promoters or enhancers [29,33].

The success of MPRA approaches depends on a variety of parameters, including the source, size, and quality of the input DNA, as well as cloning efficiency.

In this work, we obtained an enriched whole genome library of enhancer and promoter elements active in cells of an aggressive ductal adenocarcinoma pancreatic cell line MIA PaCa-2. We identified more than 11 thousand (11,264) DNA sequences representing potential enhancers and promoters in the obtained library. The mapped DNA sequences tend to be located in areas with super-enhancer properties nearby to the key genes determining a given cell type. Therefore, ChIP-lentiMPRA with inhibition of RNA interference is a useful strategy to obtain highly representative libraries of regulatory elements for the study of tumor-specific gene transcription.

## 2. Results

### 2.1. The Design of the Lentiviral System for Selection of Active Enhancers and Promoters

**Figure 1:**
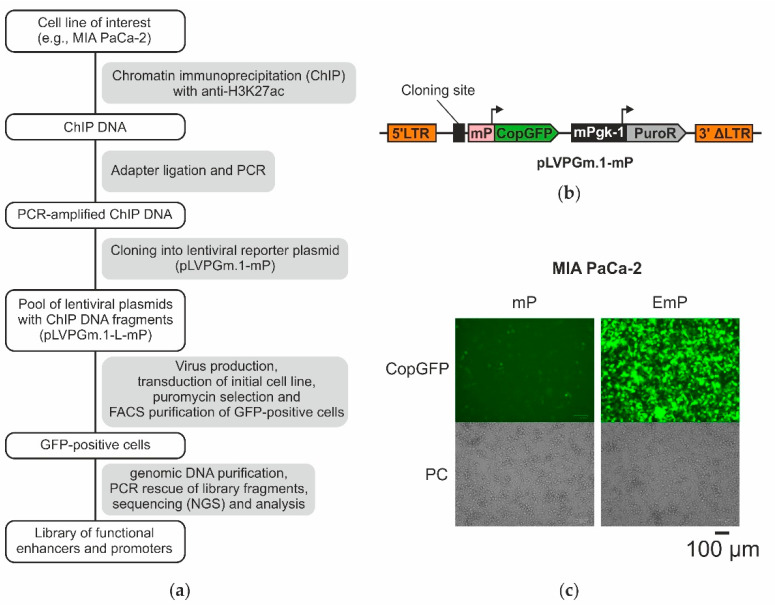
Selection of functional enhancers and promoters active in MIA PaCa-2 cells using lentiviral reporter system. (**a**) Flow chart of experimental design. We combined the method of chromatin immunoprecipitation (ChIP) and massive parallel functional assay using lentiviral reporter system (MPRA). ChIP of MIA PaCa-2 cells using H3K27ac antibody was performed for primary enrichment of the library with active enhancers and promoters. FACS was used for isolation of MIA PaCa-2 cells transduced by the lentiviral library and containing vectors with active elements. (**b**) Lentivirus vector pLVPGm.1-mP developed for the search of regulatory elements. Promoters and enhancers inserted in the cloning site should enhance activity of minimal CMV promoter in transduced cells expressing reporter gene of green fluorescent protein (CopGFP). mPgk-1-PuroR—puromycin resistance gene under control of murine Pgk-1 gene universal promoter. 5′LTR/3′ΔLTR—long terminal repeats. (**c**) Test of functionality of pLVPGm.1-mP vector. MIA PaCa-2 cells were transduced by basic vector without enhancer (mP) or by control vector containing CMV enhancer (EmP). CopGFP—fluorescence microphotography; PC—phase contrast microscopy.

We perfected the lentiMPRA technique by combining it with specific chromatin immunoprecipitation and with inhibition of RNA interference (ChIP-lentiMPRA). The general scheme for obtaining highly representative libraries of active promoters and enhancers is shown in Figure 1a.

For immunoprecipitation, we used antibodies to H3K27ac, which is the major chromatin tag of active promoters and enhancers. This allowed us to obtain an input (primary) library of ChIP DNA presumably enriched with active regulatory elements. Then we selected a fraction of the ChIP DNA fragments that are capable of enhancing the expression of the reporter gene. For this, we amplified ChIP DNA by PCR and cloned it to the lentiviral vector pLVPGm.1-mP, which was specially designed for lentiMPRA in this work. This vector contains the gene of CopGFP fluorescent protein under the control of the cytomegalovirus minimal promoter and the cloning site for enhancer analysis. pLVPGm.1-mP showed high efficiency in cloning and selection of genome regulatory elements. During cloning, we used inhibition of RNA interference by the Nodamura virus protein B2. Such an approach has been shown to dramatically increase the titer of lentiviral vectors [34]; however, it has not been previously used in the construction of libraries. After cloning, the pool of lentiviral particles was obtained, and MIA PaCa-2 cells were transduced by the virus library. Transduced cells were propagated on puromycin to eliminate cells without the vector, and then GFP-positive cells were selected using FACS. DNA from the secondary (output) library was isolated, sequenced by Next Generation Sequencing (NGS), and analyzed.

In the first stage, we constructed a basic second-generation self-inactivating lentiviral vector pLVPGm.1-mP (Figure 1b, the annotated sequence is in the Supplementary File pLVPGm.1-mP.gb). Similar vectors have been successfully used previously [31,35].

The pLVPGm.1-mP vector contains a region for cloning the DNA fragments under study in close proximity to the minimal cytomegalovirus promoter (mP) controlling the reporter gene of the fluorescent protein CopGFP (Evrogen, Russia). The vector also includes the mPgk-1-PuroR cassette, which provides transduced cells resistance to puromycin. On the basis of pLVPGm.1-mP, a positive control vector pLVPGm.1-EmP was also obtained, which contains a cytomegalovirus enhancer inserted in the cloning region near the mP promoter.

To test the functionality of the obtained lentiviral vectors, we used MIA PaCa-2 pancreatic ductal adenocarcinoma cells. MIA PaCa-2 cells transduced with a basic vector without an enhancer or a control vector containing a cytomegalovirus enhancer were obtained (Figure 1c). After puromycin selection, the cells were analyzed by flow cytofluorimetry. The cell population transduced with the enhancer control vector contained significantly more GFP-positive cells with higher fluorescence than the population transduced with the basic vector without enhancer (Figure S1). To show that the observed differences are indeed related to the activity of the promoter-enhancer pair rather than to the different number of viral integrations, we analyzed the lentiviral DNA content in the studied populations. For this purpose, genomic DNA was isolated from the cells and analyzed by quantitative PCR using primers targeted to lentiviral DNA and to a segment of genomic DNA (see Materials and Methods). The analysis showed that both samples contained comparable amounts of lentiviral DNA, corresponding to 2–3 copies per cell (Figure S2). This proves that the minimal cytomegalovirus promoter is indeed activated by the control enhancer in MIA PaCa-2 cells.

To evaluate the performance of the proposed approach, we analyzed viral titer using the FACS-based titration on MIA PaCa-2 cells (see Materials and Methods) and evaluated the dependence of transduction efficiency (percentage of transduced cells) and average lentiviral DNA copy number in transduced populations on virus dilutions during transduction (Figure S2).

At a titer of 2.3 million transducing units (TU) per ml and 15 mL of viral medium volume, we obtained about 35 million functional viral particles (TU). We showed that transduction with undiluted viral stock of the control vector (MOI 7.2 TU per cell), 61% of GFP-positive cells in the population were detected with a relatively low number of integrated lentiviral DNA of 2.3 copies per cell (Figure S2). These data indicate the high performance of the proposed approach, which can ensure the selection of functional sequences from millions of DNA fragments. Subsequently, all transduction experiments were performed using undiluted viral stocks.

### 2.2. Bidirectional Transcription as a Probable Cause of Reduced Representation of Functional Elements in Lentiviral Libraries

It was previously shown that antisense transcripts in lentiviral plasmids lead to the destruction of viral pre-mRNA in the packaging cells because of RNA interference and, consequently, to a decrease in viral titer [36]. Since enhancers and promoters often initiate transcription in the opposite direction or bidirectionally [3], we hypothesized that the production of viral particles by the pLVPGm.1-mP vector containing promoters and enhancers can also be reduced by RNA interference.

To test this assumption, we examined the production of viral particles in 293T cells transduced with an equimolar mixture of pLVPGm.1 plasmids containing the CopGFP gene under the control of a strong cytomegalovirus (CMV) promoter/enhancer in sense or antisense orientations (Figure S3). Indeed, the presence of a vector containing the CMV promoter in the antisense orientation reduces the total production of viral particles by a factor of 5.6 compared with a vector containing the CMV promoter in the forward orientation alone.

Addition of a plasmid expressing the RNA interference suppressor protein NovB2 (Nodamuravirus B2 protein) during co-transfection of the packaging cells resulted in almost complete restoration of the viral titer values, which is consistent with the results of other studies [36]. Therefore, in all experiments, a plasmid with the NovB2 gene was used for the production of viral particles in order to level out the decrease in lentiviral titer caused by RNA interference.

### 2.3. Obtaining a Primary Library Using ChIP and Cloning It into the Lentiviral Plasmid pLVPGm.1-L-mP

We performed chromatin immunoprecipitation (ChIP) of MIA PaCa-2 cells with antibodies to H3K27ac to obtain a primary DNA library enriched with promoters and enhancers. The library of ChIP DNA fragments was ligated to the adapter containing the library primer annealing site and the restriction site required for cloning (See Materials and Methods). After PCR amplification, the fragments (average length about 750 bp, Figure S4a) were cloned into the basic lentiviral plasmid pLVPGm.1-mP. Approximately 20 million colonies were obtained. PCR screening of 30 randomly selected colonies showed that they all contained inserts (Figure S4b). A pool of lentiviral plasmids pLVPGm.1-L-mP containing cloned ChIP DNA fragments was isolated, producing the *E. coli* library of 20 million clones with close to 100% clones with inserts. This indicates a high representation of ChIP DNA fragments in the pool of pLVPGm.1-L-mP plasmids.

**Figure 2:**
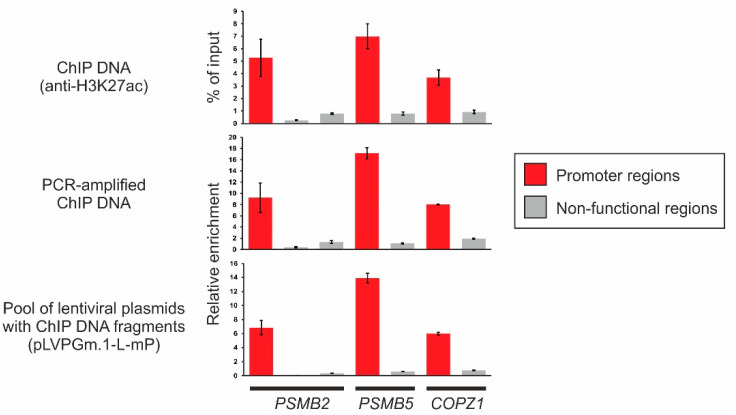
Analysis of the enrichment of ChIP DNA with functional elements using the promoter regions of the housekeeping genes *PSMB2*, *PSMB5*, and *COPZ1*. Initial ChIP DNA was immunoprecipitated from MIA PaCa-2 cells using antibodies to H3K27ac, ligated with adapters, amplified by PCR, and cloned to lentiviral plasmid pLVPGm.1-mP in *E. coli* cells. ~20 × 10^6^ clones were received, and a pool of lentiviral plasmids with inserts was isolated. Content of promoter regions of *PSMB2*, *PSMB5*, and *COPZ1* genes (red columns), as well as non-functional regions (gray columns), was determined in initial, amplified, or cloned in lentivirus ChIP DNA by qPCR. Mean of three experiments and standard deviation are presented.

Using quantitative PCR, we evaluated the enrichment of immunoprecipitated DNA with fragments of functional elements at different stages of library construction using promoter regions of the housekeeping genes *PSMB2*, *PSMB5,* and *COPZ1* (Figure 2).

As shown in Figure 2, the content of promoter fragments in the immunoprecipitated DNA exceeds four to 20 times the content of “non-functional” sites located at the same loci and not overlapping with any known ENCODE cis-regulatory elements [37] (see Materials and Methods).

It is important that despite the numerous procedures performed during the cloning of the primary ChIP DNA library (adapter ligation, PCR-amplification, restriction endonuclease digestion, ligation, multiple DNA purifications, etc.), its enrichment with functional sites did not change qualitatively.

### 2.4. Selection of Enhancers and Promoters Active in MIA PaCa-2 Cells

A pool of lentiviral plasmids pLVPGm.1-L-mP from the previous stage was used to produce viral particles in the 293T packaging cell line. After transduction and selection of transduced cells with puromycin, 12 × 10^5^ GFP-positive cells with the highest fluorescence intensity were selected using FACS. This experiment was repeated, and 25 × 10^5^ cells were obtained additionally. Genomic DNA was isolated from both populations, and preparative PCR amplification of the selected fragments was performed (see Materials and Methods). As a result, L1 and L2 libraries, presumably enriched with functional elements, were obtained from the two populations of selected cells.

**Figure 3:**
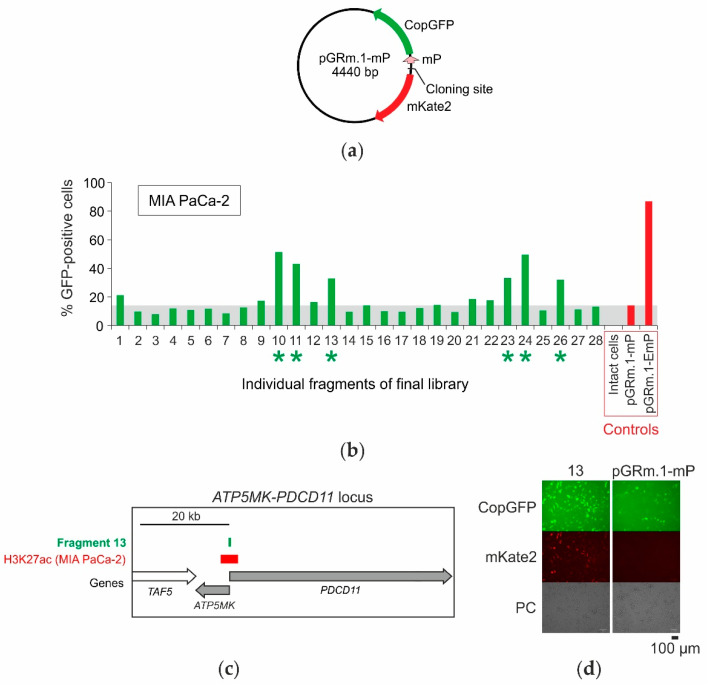
Functional analysis of individual fragments of the resulting library by the bidirectional reporter gene pair assay in transient expression system and MIA PaCa-2 cells. (**a**) Scheme of the pGRm.1-mP plasmid designed to analyze the enhancer/promoter activity of DNA fragments in transiently transfected cells. (**b**) Library of fragments isolated from GFP-positive cells was cloned into the pGRm.1-mP plasmid, twenty-eight plasmids were transfected into MIA PaCa-2 cells. Six of the 28 fragments (21%) were active in MIA PaCa-2 cells (marked with green asterisks). The gray band corresponds to the proportion of GFP-positive cells in the population transfected with the original pGRm.1-mP plasmid. pGRm.1-EmP is a control plasmid containing a CMV minimal promoter and CMV enhancer. (**c**) Fragment #13 mapped to the bidirectional promoter of the *ATP5MK* and *PDCD11* genes pair (right) and overlaps with the H3K27Ac-enriched region in Mia PaCa-2 cells. (**d**) Micrographs of MIA PaCa-2 cells transfected with pGRm.1-mP plasmid with fragment #13 and control enhancerless plasmid. PC—phase contrast.

To estimate the proportion of functional elements in the obtained libraries, the activity of 28 randomly selected DNA fragments from the L1 library was analyzed in the transient expression system. The fragments were cloned into the pGRm.1-mP plasmid (Figure 3a) 5′-end to the cytomegalovirus minimal promoter (mP) in the same orientations relative to the CopGFP gene as in the lentiviral DNA. The pGRm.1-mP plasmid contains an additional reporter gene for the red fluorescent protein mKate2, located head-to-head relative to the CopGFP gene. The second reporter gene allows the detection of dual activity, such as promoter and enhancer activity, simultaneously, as well as bidirectional promoter activity in the fragments under study. Transient transfection of MIA PaCa-2 cells was performed, and the proportion of GFP-positive cells was measured. Out of 28 DNA fragments tested, six (21%) significantly enhanced the minimal promoter activity, leading to an increase in the fraction of GFP-positive cells in the transfected populations (Figure 3b). The six active elements found were sequenced and mapped in the human genome (Table 1).

All six fragments are localized in known promoter regions and overlap with known DNase I hypersensitivity sites, ChIP-seq peaks of transcription factors [39], and ChIP-seq peaks of H3K27ac in MIA PaCa-2 cells [38]. Five fragments overlap with known transcription initiation regions (CAGE-peaks) [40]. One of the fragments (#13), mapped in the promoter region of the bidirectional gene pair *ATP5MK* and *PDCD11* (Figure 3c), enhanced the expression of both reporter genes (Figure 3d).

In our transfection experiments, just about 21% of the selected fragments contained active functional elements, and the rest seemed to be false positives. To understand the nature of the false results better, we assessed the multiplicity of lentiviral DNA integration in the L1 population using quantitative PCR (see Materials and Methods). The lentiviral DNA content in the L1 population was 2.4 copies per cell on average, which is comparable with the results obtained previously for the control populations (Figure S2). This means that the presence of a significant number of false-positive fragments in the library can be explained by multiple integrations of the lentivirus. Another source of false positives in our experiments is the so-called “position effect” that occurs when an inactive DNA fragment in the integrated lentiviral construct is influenced by a nearby genomic regulatory element (e.g., an enhancer), which activates the promoter and, consequently, the expression of CopGFP. The position effect can be overcome by non-random integration of the lentiviral construct into a certain place in the genome that is known to be not influenced by regulatory elements or isolated from them, for example, by using insulators. Unfortunately, this approach does not seem to be satisfactory because of the low efficiency of homologous recombination. This approach also ceases to be universal and requires the creation of special cell lines of each type. In principle, the position effect can be partially overcome by parallel analysis of two or more independently derived libraries through random integration of lentiviral constructs into the genome.

### 2.5. Analysis of the Distribution of Identified Fragments in the Genome

The DNA fragments of the obtained libraries were sequenced using massive parallel paired-end sequencing. The resulting reads were mapped to the human reference genome (GRCh38/hg38). In total, 6244 and 5245 fragments were mapped for the L1 and L2 libraries, respectively. Analysis of the overlap of the mapped fragments with different functional regions showed that both libraries are equally enriched in functional sites (DNase I hypersensitivity sites, transcription factor binding sites, etc.) compared to the library of random fragments obtained in silico (Table S1). Therefore, fragments from the two libraries were combined into one; overlapping fragments were merged, resulting in a map containing 11,264 unique regions (mapped regions) (Table S2).

**Figure 4:**
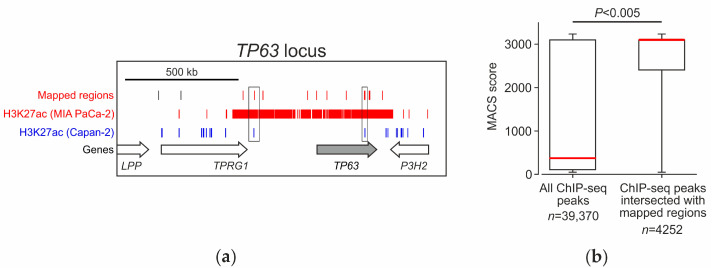
Analysis of the location of the mapped regions relative to H3K27ac-rich genomic regions. (**a**) Location of selected fragments in the vicinity of *TP63* gene expressed in Mia PaCa-2 cells and not expressed in Capan-2 cells. Mapped fragments preferentially overlap with the H3K27ac enriched regions in MIA PaCa-2 cells (highlighted in red). Fragments (2 of 14) that overlap with H3K27ac enriched regions in both MIA PaCa-2 and Capan-2 cells are boxed. (**b**) The mapped fragments tend to be localized in the MIA PaCa-2 H3K27ac-rich chromatin regions. “Box and whiskers” plot displays the distribution of ChIP-seq peaks of H3K27ac in MIA PaCa-2 by mapped read density (MACS score, Model-based Analysis of ChIP-Seq). Mapped regions overlap predominantly with regions of the genome with high H3K27ac content (Mann–Whitney test, min-max range). “*n*” is the number of ChIP-seq peaks. Red line indicates the median value.

The obtained map was compared with the maps of regions with elevated H3K27ac modification levels in MIA PaCa-2 and Capan-2 cells, as reported by Diafferia et al. [38]. Of the 11,264 regions we mapped, 5372 (~48%) overlap with high H3K27ac regions in MIA PaCa-2 cells (Tables S1 and S2). In Capan-2 cells of the epithelial pancreatic cancer subtype, the proportion of overlapping regions is slightly lower, ~41% (4651) (Table S2). For randomly selected genome fragments, the extent of overlapping with H3K27ac ChIP-seq peaks is significantly lower and does not exceed 5.7% (Table S1). These data confirm that the library obtained is enriched with sequences that overlap with the H3K27ac regions and contains, among others, elements specific to MIA PaCa-2 cells. For example, in the TP63 transcription factor gene locus, which is expressed in MIA PaCa-2 cells but is inactive in Capan-2 cells, 12 of 14 mapped fragments overlap with the H3K27ac regions in MIA PaCa-2 cells, 9 of which being specific MIA PaCa-2 (Figure 4a). At the same time, we found no fragments overlapping with Capan-2-specific H3K27ac regions, while only three fragments overlapped with H3K27ac regions present in both cell types.

The mapped fragments tend to co-localize with regions with the highest content of the H3K27ac chromatin tag (Figure 4b). High H3K27ac content is known to be characteristic of super-enhancers, which are clusters of enhancers with high cell specificity. Super-enhancers ensure high transcriptional activity of the genes they regulate, many of which are key cell identity genes [41,42]. Indeed, 8.7% of the mapped regions overlap with known MIA PaCa-2 super-enhancers, compared to only 0.8% of randomly selected genome fragments (random regions, Table S1). The mapped regions are also enriched in other features of functional chromatin traits (DNase I hypersensitivity sites, transcription factor binding sites, and CAGE peaks). For example, sequences containing transcription initiation regions (CAGE-peaks) [40] are seven times more frequently occurring among our mapped regions than among randomly selected regions of the genome (Table S1). A detailed analysis of the distribution of the mapped and randomly sampled regions in the genome showed that, indeed, the found regions are concentrated in the promoter regions of genes within ±2 kbp of their 5′-ends (Figure S5).

### 2.6. Analysis of Genes Located near Mapped Regions

As noted above, a total of 11,264 selected fragments were mapped in the genome. Our goal was to search for functionally active elements of the genome, so we further considered only the H3K27ac enriched regions. We found that the mapped regions are concentrated near gene promoters (Figure S5). We divided them into two groups: proximal (P-regions hereafter) located within ±2 kb of the 5′-end of genes and distal (D-regions) located 2–500 kb from the 5′-end of genes (Table S2).

A total of 2537 proximal and 2801 distal regions were identified. Using the GREAT service [43], we determined the association of the mapped regions with genes. GREAT associates genomic regions with genes in whose regulatory domains it lies based on data of chromosomal conformation capture approaches as well as computational methods.

**Figure 5:**
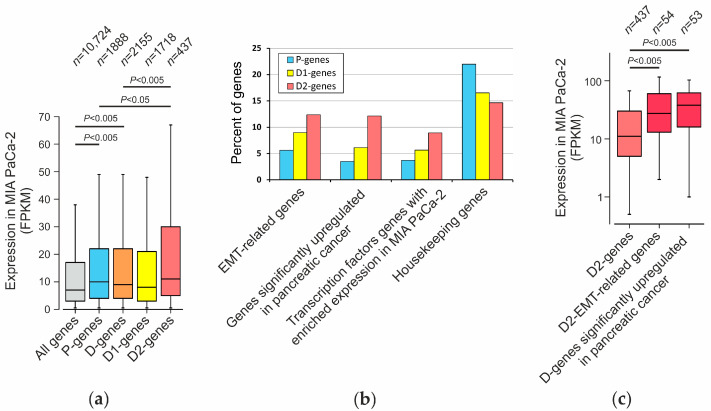
Expression of genes associated with mapped regions in MIA PaCa2 cells. Expression is presented in FPKM (fragments per kilobase of a transcript per million mapped reads). “Box and whiskers” plot shows distribution of the genes by expression levels (Mann–Whitney test, min–max range, 1.5 IQR, cut-off level 0.5 FPKM). (**a**) P-, D1- and D2 genes (see the text) were determined by GREAT (Genomic Regions Enrichment of Annotations Tool) [43], and their expression in MIA PaCa2 cells was estimated using Expression Atlas [44]. (**b**) The percentage of different functional types of genes in P- and D groups. (**c**) The most active D2 genes are EMT-related genes as well as genes with elevated expression in pancreatic cancer [45].

Using Expression Atlas data [44], we showed that the genes associated with proximal (P genes, 1888 genes) or distal (D genes, 2155 genes) regions (Table S4) are expressed at high levels in MIA PaCa-2 cells (Figure 5a and Table S4). This means that the regions we mapped tend to localize near the most active genes and that the libraries we obtained are enriched with elements that are probably involved in the regulation of these genes.

We divided the D genes into genes associated with 1–2 mapped regions (D1 genes, 1718 genes) and genes associated with three or more distal regions (D2 genes, 437 genes) (Table S4). Interestingly, the D2 group genes are, on average, expressed at a higher level than the P and D1 genes (Figure 5a). We also found that gene groups (P-, D1-, and D2 genes) differ from each other in the content of genes of different functional categories (Figure 5b). In particular, we distinguish genes involved in epithelial–mesenchymal transition (EMT-related genes) [46], genes significantly upregulated in pancreatic cancer [45], and genes of transcription factors showing increased expression in MIA PaCa-2 cells [47].

The vast majority of genes belong to one of the three categories reviewed, though some genes may belong to more than one category (Figure S6). As shown in Figure 5b, the proportion of genes related to these three categories is higher in gene group D2 than in gene group D1 and especially than in gene group P. At the same time, the content of the “housekeeping” genes [48] is higher among P genes. These results are consistent with the data of Zabidi et al., 2015 [49] that enhancers of “housekeeping” genes are more often concentrated near the transcription start sites (TSS) of genes than enhancers of genes with complex and tissue-specific regulation.

D2-genes of the categories “EMT-related genes” (54 genes) and “Genes significantly upregulated in pancreatic cancer” (53 genes) are, on average, expressed at higher levels in MIA PaCa-2 cells than other genes in this group (Figure 5c and Table S4). Many of the identified D2 genes encode transcription factors with the expression characteristic for MIA PaCa-2 cells, such as *HMGA1*, *MYC*, *ID1*, *NR2F1*, *CEBPB*, *STAT3*, *TP63*, *SOX9*, *ZEB1,* and others [47]. These results indicate that the obtained library of DNA fragments contains specific enhancer-like (regulatory, promoter, and enhancer) elements that are involved in the regulation of key identity genes that determine the individual type of MIA PaCa-2 cells.

**Figure 6:**
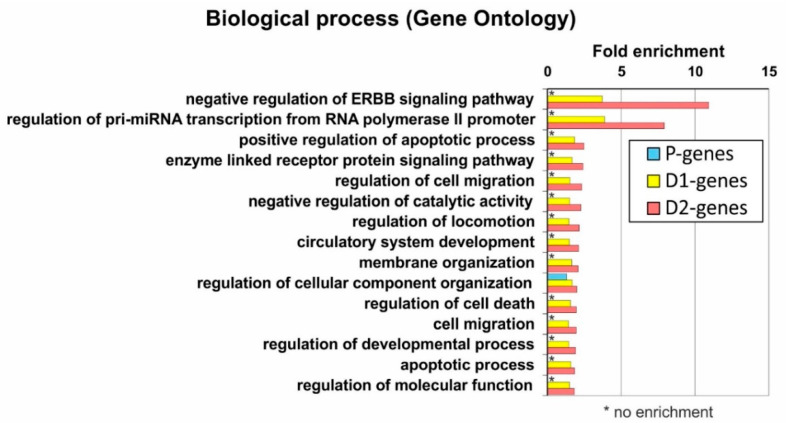
Enrichment analysis of GO-terms (biological process) characteristic for D2 genes for all groups of genes (D1-, D2-, and P genes). Fold enrichment was determined using DAVID [54].

For a more detailed analysis of the functions of the genes associated with the mapped regions, we investigated their features using the Gene Ontology database. We showed that the D2 gene group is enriched in genes that are involved in processes relevant to carcinogenesis, in particular, in the regulation of cell death (GO:0010941) and apoptosis (GO:0043065) [50], cell migration and motility (GO:0030334; GO:0040012) [51], and ERBB-linked signaling pathway (GO:1901185) [52,53] (Figure S7a). The D1 group has significantly fewer genes associated with these processes, and they are virtually absent among the P genes (Figure 6). At the same time, the group of P genes associated with proximal regions is enriched in genes involved in fundamental processes typical for housekeeping genes—regulation of DNA metabolism (GO:0051052) and chromosome organization (GO:0033044), cell cycle processes (GO:0022402; GO:0010564), responses to virus transcription (GO:0019083), DNA damage (GO:0006974) and others (Figure S7b). Thus, the gene repertory defined by Gene Ontology is consistent with the result obtained by direct analysis of gene groups (see above and Figure 5).

## 3. Discussion

Pancreatic cancer is one of the most aggressive and malignant tumors. As with other tumors, successful treatment requires early diagnosis, correct subtyping, knowledge of general patterns of the disease, and morphological and molecular characterization of a particular case [55,56,57,58]. In this regard, it is of great importance to study both general and specific patterns of this type of cancer, peculiarities of expression of molecular markers, and master regulators of gene expression. The creation of DNA libraries of active genome regulatory elements and the development of new methods for creating such libraries contribute to the study of the pathogenesis of pancreatic cancer.

In this work, we constructed a library of active genome regulatory elements (promoters and enhancers) from MIA PaCa-2 pancreatic ductal carcinoma cells using an improved approach combining chromatin immunoprecipitation with a lentivirus vector-based massive parallel reporter assay (ChIP-lentiMPRA).

The initial set of DNA fragments for cloning was obtained by immunoprecipitation of MIA PaCa-2 cell chromatin with antibodies to the acetylated histone H3K27ac. This chromatin modification represents one of the most important but not the only characteristic of active enhancers [1], which is probably necessary for enhancer RNA transcription [59]. Compared to MPRA variants that used fragmented genomic DNA [31,60] or synthesized DNA [29,32,33,35], preliminary immunoprecipitation of chromatin is beneficial because it immediately enriches the original cell DNA with prospective active regulatory elements, including unknown ones. Additionally, immunoprecipitation allows to eliminate silent genome elements such as long terminal repeats (LTRs) which may have strong, usually non-specific promoter and/or enhancer activities outside of their native context.

The isolated H3K27ac-ChIP DNA fraction was cloned into the lentiviral vector pLVPGm.1-mP, which we constructed for efficient cloning and selection of active promoters and enhancers. This vector contains a CopGFP reporter gene under the control of the minimal CMV promoter and a site for cloning the DNA elements under study. Earlier, other authors used lentivirus vectors in the creation of libraries for MPRA [29,31,32,33]. Lentiviruses integrate into the cell genome and produce stable transfectants. This is significantly advantageous compared to episomal [32] or BAC-based [61] analyses because lentivirus vectors provide an in-genome read-out of the candidate regulatory sequences under study. Lentiviruses can infect many different cells and tissues, including those that may hardly be transfected by episomal vectors. Therefore, lentiMPRA allows to functionally test thousands of prospective promoters or enhancers in many biological systems. The drawbacks of lentiMPRA stem out of lentivirus advantage to integrate into cell genome because of random integration. Thus, adjoining chromosomal landscape, as well as variable integration multiplicity, may influence the efficiency of the regulatory sequences under study significantly. In our experiments, we demonstrate that MIA PaCa-2 cells transduced by pLVPGm.1-mP with or without enhancer contain 2–3 virus copies per cell.

In addition, we applied inhibition of RNA interference by the Nodamura virus protein B2 (NovB2) [34], which has not been used previously in the construction of libraries. An initial evaluation showed that this approach allows the production of DNA libraries containing up to 34.5 million clones, and the use of NovB2 effectively prevents a drop in the production of viral library particles due to RNA interference.

As a result, the *E. coli* library of immunoprecipitated DNA fragments we obtained from MIA PaCa-2 cells contained about 20 million clones. Up to 100% of the clones contained a vector with an insert, with at least 21% of the inserts representing active functional genomic elements.

Massive parallel sequencing of the clones obtained after FACS of the primary library allowed us to identify 11,264 unique genome regions enriched with the H3K27ac tag characteristic of promoters, enhancers, and super-enhancers. The result obtained significantly exceeds the number of regulatory elements previously studied by other authors in MRPA, where the initial DNA was obtained by other methods. For example, in the work of Inoue et al. [29], 2464 pre-synthesized enhancers were studied; Murtha et al. [31] identified approximately 6300 enhancers among 80,000 cloned DNA fragments of nucleosome-free regions.

Among the genes associated with the regions mapped in this work, we identified genes related to epithelial–mesenchymal transition and a group of genes for which increased expression has been shown in pancreatic cancer. Most genes of these groups are associated with three or more mapped distal regions (D2 genes). This may indicate a relationship between the density of enhancer-like elements and the expression levels of genes associated with them. We also identified genes encoding transcription factors specific to the cell line under study—*HMGA1*, *MYC*, *ID1*, *NR2F1*, *CEBPB*, *STAT3*, *TP63*, *SOX9*, and others [47]. The association with the genes of these groups is most significant for the mapped regions that are located at distances of 2–500 kbp from the 5′-terminus of the genes and are associated with 3 or more different genes. Among these genes, a significant proportion is represented by genes that are involved in processes relevant to carcinogenesis, such as regulation of cell death and apoptosis, cell migration, cell motility, and the ERBB-linked signaling pathway.

The distribution of mapped regions is consistent with the findings of other researchers that many enhancers of “housekeeping” genes tend to concentrate near the 5′-ends of genes, while enhancers of genes with complex specific regulation (such as those regulating organism development) are more often located at a considerable distance from the 5′-ends of genes, in introns or intergenic space [49]. In addition, we previously showed that the tumor-specific expression of some genes involved in the regulation of cell division is caused by sequences no more than 0.5 kbp distant from the 5′-ends of genes [62]. Apparently, both cis- and trans-regulatory elements are usually involved in the regulation of the expression of a number of genes related to carcinogenesis, with housekeeping genes often restricted to cis-regulation. This can be explained by both the evolutionary antiquity and conservatism of housekeeping genes, as well as the lack of the need for complex or conditional regulation of their expression.

Thus, our approach to the construction of a library of functional genome elements and the principle of selection of cloned DNA fragments allowed us to identify important genome regulatory elements available for further studies. Many of these elements may act like master regulators because they are associated with groups of genes (three or more genes) that are responsible for tumorigenic or tumor-like cell behavior. Mutations of regulatory elements or changes in their epigenetic landscape due to various reasons can contribute to tumor progression even without damaging the target genes themselves [63,64].

It should be noted that histones on active regulatory elements can also carry other epigenetic tags, including both acetyl (H3K64ac and H3K122ac) and methyl groups (H3K4Me1), as well as having low H3K27ac [21,65]. In this study, immunoprecipitation was performed with antibodies to H3K27ac. Therefore, our isolated regulatory elements were enriched only with those carrying the corresponding histone. Perhaps immunoprecipitation with antibodies to several histone modifications should be used to isolate more active regulatory elements. It would be interesting to compare functionally the sets of genes regulated by active enhancers characterized by different epigenetic markers, such as H3K27ac+ and H3K27ac-/H3K64ac enhancers [21], and the technology we used allows us to do so. Further study of cloned enhancers and promoters, as well as the construction of libraries of genome regulatory elements based on other principles, will allow us to understand the specific role of such elements in cellular processes, including the processes of carcinogenesis.

## 4. Materials and Methods

A detailed description of the experimental design can be found in the methods in Supplementary Materials.

### 4.1. ChIP DNA Preparation and Cloning

Approximately 1 × 10^7^ logarithmically growing MIA PaCa-2 cells were fixed, pelleted, and lysed as detailed in the methods in Supplementary Materials. The lysate was sonicated with a Cole-Parmer CP750 ultrasonic processor and precleared with Dynabeads Protein G (Life Technologies, Carlsbad, CA, USA). The lysate was incubated with anti-H3K27ac antibodies (Abcam, ab4729, Cambridge, UK), and immune complexes were sorbed on Dynabeads Protein G, sedimented, and eluted from the beads. ChIP DNA and Input DNA were extracted using a PCR purification kit (Qiagen, 28104, Hilden, Germany). Aliquots were used to assess ChIP DNA enrichment by functional elements relative to Input DNA by quantitative real-time PCR (qPCR). For this, promoter and non-promoter regions from three loci of housekeeping genes *PSMB5*, *PSMB2,* and *COPZ1* were amplified. Non-promoter regions were selected from the regions that do not overlap with potential cis-regulatory elements (UCSC Browser track “ENCODE Candidate Cis-Regulatory Elements (cCREs) combined from all cell types”). Primers are listed in Table S5.

### 4.2. Lentivirus Production

293T cells were transfected with 12 μg lentiviral plasmid, 6 μg pMD.G [66], 12 μg pCMV∆R8.91 [67], and 7.5 μg pcDNA3.1 puro Nodamura B2 (Addgene, Watertown, MA, USA, cat.#17228 [34]) using Lipofectamine 2000 (Invitrogen, Waltham, MA, USA) according to the manufacturer’s recommendations. Cells were incubated, and the virus was collected from a culture medium. Virus stocks were immediately used for MIA PaCa-2 cell transduction.

### 4.3. FACS-Based Titration of Control Lentiviral Vector pLVPGm.1-EmP

The MIA PaCa-2 cells were transduced with both non-diluted virus and with dilutions 1/32, 1/64, 1/128, and 1/256. After incubation, the viral-containing medium was replaced with a fresh medium. Then, the numbers of GFP-positive cells in each well were determined by flow cytometry, and the titer was calculated using a percentage of GFP-positive cells.

### 4.4. Adapter Ligation and PCR

The End Repair/dA-Tailing Module (NEB, E7442S, Ipswich, MA, USA) was used to convert ChIP DNA to repaired DNA having 5’ phosphorylated dA-tailed ends. Repaired DNA fragments were ligated to adapter, obtained by annealing equimolar amounts of the two oligonucleotides (oligo 1; oligo 2; Table S5). After ligation, the DNA was purified, combined with library primer (Table S5), and amplified. An aliquot was used to assess amplified library enrichment by functional elements relative to Input DNA by qPCR.

### 4.5. ChIP DNA Cloning

PCR-amplified ChIP DNA was digested with Xho I restriction endonuclease and ligated into XhoI- and SalI-treated pLVPGm.1-mP. The ligated vector was used for transformation of DH5-alpha *E. coli*, and pool of plasmid DNAs (pLVPGm.1-mP-L) with ChIP DNA library fragments was isolated. An aliquot of the library was used to assess cloned ChIP DNA enrichment by functional elements relative to Input DNA qPCR as described above. In total, 30 colonies grown on agar plates were analyzed by PCR with primers P1 and P2 (Table S5) for the presence of inserts with primers flanking inserted DNA fragments.

### 4.6. Massive Functional Selection of Enhancers and Promoters, Active in MIA PaCa-2 Cells

#### 4.6.1. Transduction of MIA PaCa-2 Cells

Pool of lentiviral viral particles was used for the transduction of MIA PaCa-2 cells. GFP-positive cells with higher fluorescence signal were selected using BD FACSAria III (BD Biosciences, Haryana, India) cell sorter, and approximately 3.7 × 10^4^ of transduced GFP-positive cells were collected.

#### 4.6.2. PCR Amplification of Inserts and Functional Assay

Genomic DNA was isolated from GFP-positive cells and amplified by nested PCR using primers P3, P4, and nested primers P1 and P2 (Table S5). Amplified DNA was cut by XhoI and SbfI restriction enzymes and ligated into pGRm.1-mP plasmid by the same restriction sites. *E. coli* DH-5alpha was transformed by the ligated vector. Plasmid DNAs containing inserts were isolated from 28 clones and used for transfection of MIA PaPa-2 cells. Populations of transfected cells were analyzed by a Nikon TE2000U microscope equipped with epifluorescence optics (Nikon Europe, Amsterdam, The Netherlands) and flow cytofluorimeter FACScan (BD Biosciences).

#### 4.6.3. Libraries Preparation and Next-Generation Sequencing

Samples of genomic DNA isolated from GFP-positive clones were amplified using primers and conditions recommended by Illumina protocol “16S Metagenomic Sequencing Library Preparation” (Illumina, San Diego, CA, USA). The first PCR was performed using external primers P3 and P4 (Table S5), flanking the lentivirus-cloned fragments. The second PCR was performed using internal primers, R1M15f and R2M15r (Table S5), that flank fragments cloned into lentivirus and contained sections of Illumina adaptor sequences required for paired-end sequencing. The third PCR was performed using standard Illumina primers, P5S502 and P7N70X (Table S5), containing additional adaptor sequences required for paired-end sequencing. The P7N70X primer contained 8 bp Index sequence unique to each resulting library. Libraries of DNA fragments ranging from 400 to 1400 bp in length were purified using agarose gel electrophoresis, mixed, and sequenced on Illumina MiSeq at both ends (2 × 150 bp).

#### 4.6.4. Paired-End Reads Mapping

R1 and R2 files of each library were transformed to fastqsanger format using FASTQ Groomer (Galaxy Version 1.0.4) (Input FASTQ quality scores type: Sanger & Illumina 1.8+; advanced options: basic) [68]. Adapter sequencers were eliminated with Cutadapt. The ends of the reads were trimmed with FASTQ Trimmer (Galaxy Version 1.1.5) [68]. Paired-end reads were mapped using the human genome hg38 as a reference with Bowtie2 (Galaxy Version 2.2.6.2) [69]. The reads were processed with Filter Bam datasets on a variety of attributes (Galaxy Version 2.4.1) [70]. First, only properly paired reads corresponding to primary alignments with MAPQ ≥ 10 were kept, then reads from the different DNA chains were separated using alignment flag combinations (99 or 147 for the plus strand and 83 or 163 for the minus strand). Files containing reads were converted into BEDPE format with BAM to BED converter from BedTools (Galaxy Version 2.30.0) [71]. Overlapping regions of the same orientation were merged (Galaxy Version 1.0.0), and obtained BED files were converted to GFF format. The RPM (reads per million) values were calculated by htseq-count (Galaxy Version 0.9.1) [72]. GFF files containing regions with different orientations were combined, and regions with RPM values greater than 10 were used for further analysis.

### 4.7. Bioinformatic Data Analysis

We analyzed the intersection of mapped regions and random genomic regions with known ChIP-seq H3K27ac peaks obtained for MIA PaCa-2 and Capan-2 cells [38], with with “DNase I Hypersensitivity peak clusters” (95 cell types) and “Transcription Factor ChIP-seq Clusters” (340 factors, 129 cell types) from ENCODE [73], with known super-enhancers from the SEA database [74], with MIA PaCa-2 super-enhancers from SEdb 2.0 [75] and CAGE peaks from FANTOM5 project [40]. ChIP-seq peaks of H3K27ac for MIA PaCa-2 and Capan-2 lines as BED files with accessions GSM1574239 and GSM1574236, respectively, were downloaded from the NCBI GEO database [76]. BED files with ENCODE data were downloaded using the Table Browser tool of the UCSC genomic browser (http://genome.ucsc.edu (accessed on 7 April 2022)). A BED file with human 93,873 super-enhancers derived from H3K27ac ChIP-seq data for various cell types was downloaded from the SEA version 3.0 database (http://sea.edbc.org (accessed on 7 April 2022)). The coordinates of the MIA PaCa-2 super-enhancers were extracted from file SE_02_0723_SE_hg38.bed (hg38), downloaded from the SEdb 2.0 database (http://www.licpathway.net/sedb (accessed on 21 November 2022)). Coordinates of the CAGE peaks were obtained using the Table Extraction Tool of the FANTOM5 project (https://fantom.gsc.riken.jp/5/ (accessed on 7 April 2022)). Random genomic regions were generated using the ShuffleBed tool [71] on the web-based Galaxy platform (https://usegalaxy.org (accessed on 7 April 2022)) [77]. The number of random regions, their size, and chromosome distribution are in perfect accordance with those for the mapped regions. Intersections of the mapped regions and random regions with the genomic intervals of the downloaded datasets were determined using the tool “Intersect the intervals of two datasets” on the Galaxy platform.

The program SeqMonk version 1.48.1 (https://www.bioinformatics.babraham.ac.uk/projects/seqmonk/ (accessed on 7 April 2022)) was used to analyze and obtain data on the relative distribution of mapped regions around the 5-ends of genes.

### 4.8. Gene Ontology Annotation

GREAT [43] was used to identify associations between mapped regions and genes by applying the following rules: basal+extension: 2000 bp upstream, 2000 bp downstream, 500,000 bp max extension, curated regulatory domains excluded. To perform functional annotation of gene sets with Gene Ontology (GO), we used DAVID [54] (version 6.8, https://david.ncifcrf.gov/home.jsp (accessed on 7 April 2022). Obtained GO terms with corresponding p-adjusted values (*p*-adj < 0.05) were used to visualize GO terms clusters with REVIGO [78] (http://revigo.irb.hr/ (accessed on 7 April 2022)) using the following options: remove obsolete GO terms, choose species Homo sapiens (9606), SimRel (default) semantic similarity measure.

## 5. Conclusions

Our study demonstrated that ChIP-lentiMPRA approach using pLVPGm.1-mP lentiviral vector with the RNA interference inhibition is a useful strategy to create representative libraries of regulatory elements of the genome. Further study of cloned enhancers and promoters from MIA PaCa-2 cells, as well as the construction of libraries of regulatory elements based on other principles, will allow us to understand the specific role of such elements in cellular processes including carcinogenesis.

## Figures and Tables

**Table 1:** Sequenced and mapped DNA fragments from L1 library.

#	Genome Position (hg38)	Length, bp	Position Relative to Genes	DNase I HS ^1^	TFBS ^2^	SEA SE ^3^	H3K27ac ^4^	CAGE ^5^	Downstream Gene ^6^	GeneHancer
10	chr13:99,498,575- 99,499,229	655	LINC01232 and TM9SF2 promoter	yes	yes	-	yes	-	LINC01232	GH04J041933
11	chr4:41,934,622- 41,935,299	678	ENSG00000249771 and TMEM33 promoter	yes	yes	-	yes	4	TMEM33	GH04J041933
13	chr10:103,396,330- 103,396,860	531	ATP5MK and PDCD11 promoter	yes	yes	-	yes	3	PDCD11	GH10J103394
23	chr6:26,171,938- 26,172,533	596	ENSG00000283064 and H2BC6 1st exons	yes	yes	yes	yes	2	ENSG00000283064	GH06J026170
24	chr2:20,822,774- 20,823,142	369	LDAH promoter	yes	yes	-	yes	2	LDAH	GH02J020821
26	chr3:9,791,789- 9,792,567	779	TADA3 and ARPC4/ARPC4-TTLL3 promoter	yes	yes	-	yes	4	ARPC4/ARPC4-TTLL	GH03J009791

^1^ DNase I hypersensitivity peak clusters from ENCODE (95 cell types); ^2^ Transcription factor ChIP-seq clusters (340 factors, 129 cell types) from ENCODE 3; ^3^ Super-enhancers from SEA database (http://sea.edbc.org/ (accessed on 7 April 2022)); ^4^ H3K27ac ChIP-seq peaks in MIA PaCa-2 cell line [38]; ^5^ CAGE peaks from FANTOM5 project (https://fantom.gsc.riken.jp/5/ (accessed on 7 April 2022)); ^6^ Downstream gene (possible target) of the DNA fragment in the genome.

## Data Availability

The NGS data obtained in this study can be found at https://disk.yandex.ru/d/xB-nGQbDsRoT3A (accessed on 24 November 2022) or can be obtained on request from the corresponding author.

## Supplementary Materials

The following supporting information can be downloaded at: https://www.mdpi.com/article/10.3390/ijms232315011/s1, Figure S1: FACS analysis of MIA PaCa-2 cells transduced by basic (pLVPGm.1-mP) or control (pLVPGm.1-EmP) vectors; Figure S2: Determination of number of copies of integrated lentiviral DNA in populations of transduced MIA PaCa-2 cells after puromycin selection; Figure S3: Inhibition of RNA-interference revokes decrease in lentivirus titer due to antisense transcription from lentiviral plasmid in transfected packing 293T cells; Figure S4: Preparation of the pLVPGm.1-L-mP lentiviral clone library; Figure S5: The mapped regions are concentrated near gene promoters; Figure S6: Representation of genes of the three functional categories among the genes associated with all the mapped regions; Figure S7: Functional annotation of genes associated with mapped regions; Table S1: Proportion of mapped fragments of the studied DNA libraries that overlap with various functional sites in the genome; Table S2: Intersections of mapped regions with some chromatin features and their associations with genes according to GREAT (http://great.stanford.edu/great (accessed on 7 April 2022)); Table S3: Proportion of mapped regions overlapping with different functional regions in the genome; Table S4: Genes active in MIA PaCa-2 cells (≥0.5 FPKM) and their associations with H3K27ac-rich mapped regions (H3K27ac+ MRs) according to GREAT (http://great.stanford.edu/great (accessed on 7 April 2022)); Table S5. Oligonucleotides used in the work; pLVPGm.1-mP.gb.

## Abbreviations

ChIP, chromatin immunoprecipitation; MPRA, massive parallel reporter assay; TSS, transcription start site; CMV, cytomegalovirus; mP, minimal promoter; EmP, minimal promoter with enhancer.

## References

[1] Ibragimov A.N., Bylino O.V., Shidlovskii Y.V. (2020). Molecular Basis of the Function of Transcriptional Enhancers. Cells.

[2] Haberle V., Stark A. (2018). Eukaryotic core promoters and the functional basis of transcription initiation. Nat. Rev. Mol. Cell Biol..

[3] Kim T.K., Shiekhattar R. (2015). Architectural and Functional Commonalities between Enhancers and Promoters. Cell.

[4] Andersson R., Sandelin A. (2020). Determinants of enhancer and promoter activities of regulatory elements. Nat. Rev. Genet..

[5] Dao L.T.M., Spicuglia S. (2018). Transcriptional regulation by promoters with enhancer function. Transcription.

[6] Wu X., Sharp P.A. (2013). Divergent transcription: A driving force for new gene origination?. Cell.

[7] Carelli F.N., Liechti A., Halbert J., Warnefors M., Kaessmann H. (2018). Repurposing of promoters and enhancers during mammalian evolution. Nat. Commun..

[8] Andersson R., Gebhard C., Miguel-Escalada I., Hoof I., Bornholdt J., Boyd M., Chen Y., Zhao X., Schmidl C., Suzuki T. (2014). An atlas of active enhancers across human cell types and tissues. Nature.

[9] Li W., Notani D., Rosenfeld M.G. (2016). Enhancers as non-coding RNA transcription units: Recent insights and future perspectives. Nat. Rev. Genet..

[10] Sartorelli V., Lauberth S.M. (2020). Enhancer RNAs are an important regulatory layer of the epigenome. Nat. Struct. Mol. Biol..

[11] Didych D.A., Shamsutdinov M.F., Smirnov N.A., Akopov S.B., Monastyrskaya G.S., Uspenskaya N.Y., Nikolaev L.G., Sverdlov E.D. (2013). Human PSENEN and U2AF1L4 genes are concertedly regulated by a genuine bidirectional promoter. Gene.

[12] Kashkin K.N., Sverdlov E.D. (2016). Properties, functions, and therapeutic prospects of enhancer RNAs. Russ. J. Bioorg. Chem..

[13] Kowalczyk M.S., Hughes J.R., Garrick D., Lynch M.D., Sharpe J.A., Sloane-Stanley J.A., McGowan S.J., De Gobbi M., Hosseini M., Vernimmen D. (2012). Intragenic enhancers act as alternative promoters. Mol. Cell.

[14] Skvortsova Y.V., Kondratieva S.A., Zinovyeva M.V., Nikolaev L.G., Azhikina T.L., Gainetdinov I.V. (2016). Intragenic Locus in Human PIWIL2 Gene Shares Promoter and Enhancer Functions. PLoS ONE.

[15] Li G., Ruan X., Auerbach R.K., Sandhu K.S., Zheng M., Wang P., Poh H.M., Goh Y., Lim J., Zhang J. (2012). Extensive promoter-centered chromatin interactions provide a topological basis for transcription regulation. Cell.

[16] Engreitz J.M., Haines J.E., Perez E.M., Munson G., Chen J., Kane M., McDonel P.E., Guttman M., Lander E.S. (2016). Local regulation of gene expression by lncRNA promoters, transcription and splicing. Nature.

[17] Paralkar V.R., Taborda C.C., Huang P., Yao Y., Kossenkov A.V., Prasad R., Luan J., Davies J.O., Hughes J.R., Hardison R.C. (2016). Unlinking an lncRNA from Its Associated cis Element. Mol. Cell.

[18] Kundaje A., Meuleman W., Ernst J., Bilenky M., Yen A., Heravi-Moussavi A., Kheradpour P., Zhang Z., Wang J., Roadmap Epigenomics Consortium (2015). Integrative analysis of 111 reference human epigenomes. Nature.

[19] Zhu Y., Sun L., Chen Z., Whitaker J.W., Wang T., Wang W. (2013). Predicting enhancer transcription and activity from chromatin modifications. Nucleic Acids Res..

[20] Stueve T.R., Marconett C.N., Zhou B., Borok Z., Laird-Offringa I.A. (2016). The importance of detailed epigenomic profiling of different cell types within organs. Epigenomics.

[21] Pradeepa M.M. (2017). Causal role of histone acetylations in enhancer function. Transcription.

[22] Nagy G., Daniel B., Jonas D., Nagy L., Barta E. (2013). A novel method to predict regulatory regions based on histone mark landscapes in macrophages. Immunobiology.

[23] Calo E., Wysocka J. (2013). Modification of enhancer chromatin: What, how, and why?. Mol. Cell.

[24] Weber C.M., Henikoff S. (2014). Histone variants: Dynamic punctuation in transcription. Genes Dev..

[25] Inoue F., Ahituv N. (2015). Decoding enhancers using massively parallel reporter assays. Genomics.

[26] Dailey L. (2015). High throughput technologies for the functional discovery of mammalian enhancers: New approaches for understanding transcriptional regulatory network dynamics. Genomics.

[27] Trauernicht M., Martinez-Ara M., van Steensel B. (2020). Deciphering Gene Regulation Using Massively Parallel Reporter Assays. Trends Biochem. Sci..

[28] Santiago-Algarra D., Dao L.T.M., Pradel L., Espana A., Spicuglia S. (2017). Recent advances in high-throughput approaches to dissect enhancer function. F1000Research.

[29] Inoue F., Kreimer A., Ashuach T., Ahituv N., Yosef N. (2019). Identification and Massively Parallel Characterization of Regulatory Elements Driving Neural Induction. Cell Stem Cell.

[30] Chernov I., Stukacheva E., Akopov S., Didych D., Nikolaev L., Sverdlov E. (2008). A new technique for selective identification and mapping of enhancers within long genomic sequences. BioTechniques.

[31] Murtha M., Tokcaer-Keskin Z., Tang Z., Strino F., Chen X., Wang Y., Xi X., Basilico C., Brown S., Bonneau R. (2014). FIREWACh: High-throughput functional detection of transcriptional regulatory modules in mammalian cells. Nat. Chem. Biol..

[32] Inoue F., Kircher M., Martin B., Cooper G.M., Witten D.M., McManus M.T., Ahituv N., Shendure J. (2017). A systematic comparison reveals substantial differences in chromosomal versus episomal encoding of enhancer activity. Genome Res..

[33] Gordon M.G., Inoue F., Martin B., Schubach M., Agarwal V., Whalen S., Feng S., Zhao J., Ashuach T., Ziffra R. (2020). lentiMPRA and MPRAflow for high-throughput functional characterization of gene regulatory elements. Nat. Protoc..

[34] Sullivan C.S., Ganem D. (2005). A virus-encoded inhibitor that blocks RNA interference in mammalian cells. J. Virol..

[35] Schlabach M.R., Hu J.K., Li M., Elledge S.J. (2010). Synthetic design of strong promoters. Proc. Natl. Acad. Sci. USA.

[36] Maetzig T., Galla M., Brugman M.H., Loew R., Baum C., Schambach A. (2010). Mechanisms controlling titer and expression of bidirectional lentiviral and gammaretroviral vectors. Gene Ther..

[37] Consortium E.P., Moore J.E., Purcaro M.J., Pratt H.E., Epstein C.B., Shoresh N., Adrian J., Kawli T., Davis C.A., Dobin A. (2020). Expanded encyclopaedias of DNA elements in the human and mouse genomes. Nature.

[38] Diaferia G.R., Balestrieri C., Prosperini E., Nicoli P., Spaggiari P., Zerbi A., Natoli G. (2016). Dissection of transcriptional and cis-regulatory control of differentiation in human pancreatic cancer. EMBO J..

[39] Rosenbloom K.R., Armstrong J., Barber G.P., Casper J., Clawson H., Diekhans M., Dreszer T.R., Fujita P.A., Guruvadoo L., Haeussler M. (2015). The UCSC Genome Browser database: 2015 update. Nucleic Acids Res..

[40] Forrest A.R., Kawaji H., Rehli M., Baillie J.K., de Hoon M.J., Haberle V., Lassmann T., Kulakovskiy I.V., Lizio M., The FANTOM Consortium and the RIKEN PMI and CLST (DGT) (2014). A promoter-level mammalian expression atlas. Nature.

[41] Whyte W.A., Orlando D.A., Hnisz D., Abraham B.J., Lin C.Y., Kagey M.H., Rahl P.B., Lee T.I., Young R.A. (2013). Master transcription factors and mediator establish super-enhancers at key cell identity genes. Cell.

[42] Hnisz D., Abraham B.J., Lee T.I., Lau A., Saint-Andre V., Sigova A.A., Hoke H.A., Young R.A. (2013). Super-enhancers in the control of cell identity and disease. Cell.

[43] McLean C.Y., Bristor D., Hiller M., Clarke S.L., Schaar B.T., Lowe C.B., Wenger A.M., Bejerano G. (2010). GREAT improves functional interpretation of cis-regulatory regions. Nat. Biotechnol..

[44] Petryszak R., Keays M., Tang Y.A., Fonseca N.A., Barrera E., Burdett T., Fullgrabe A., Fuentes A.M., Jupp S., Koskinen S. (2016). Expression Atlas update--an integrated database of gene and protein expression in humans, animals and plants. Nucleic Acids Res..

[45] Goonesekere N.C., Wang X., Ludwig L., Guda C. (2014). A meta analysis of pancreatic microarray datasets yields new targets as cancer genes and biomarkers. PLoS ONE.

[46] Zhao M., Kong L., Liu Y., Qu H. (2015). dbEMT: An epithelial-mesenchymal transition associated gene resource. Sci. Rep..

[47] Abugessaisa I., Shimoji H., Sahin S., Kondo A., Harshbarger J., Lizio M., Hayashizaki Y., Carninci P., Forrest A., The FANTOM consortium (2016). FANTOM5 transcriptome catalog of cellular states based on Semantic MediaWiki. Database.

[48] Chang C.W., Cheng W.C., Chen C.R., Shu W.Y., Tsai M.L., Huang C.L., Hsu I.C. (2011). Identification of human housekeeping genes and tissue-selective genes by microarray meta-analysis. PLoS ONE.

[49] Zabidi M.A., Arnold C.D., Schernhuber K., Pagani M., Rath M., Frank O., Stark A. (2015). Enhancer-core-promoter specificity separates developmental and housekeeping gene regulation. Nature.

[50] Ferrer C.C., Berthenet K., Ichim G. (2021). Apoptosis–Fueling the oncogenic fire. FEBS J..

[51] Yamaguchi H., Wyckoff J., Condeelis J. (2005). Cell migration in tumors. Curr. Opin. Cell Biol..

[52] Esteban-Villarrubia J., Soto-Castillo J.J., Pozas J., San Roman-Gil M., Orejana-Martin I., Torres-Jimenez J., Carrato A., Alonso-Gordoa T., Molina-Cerrillo J. (2020). Tyrosine Kinase Receptors in Oncology. Int. J. Mol. Sci..

[53] Kontomanolis E.N., Koutras A., Syllaios A., Schizas D., Mastoraki A., Garmpis N., Diakosavvas M., Angelou K., Tsatsaris G., Pagkalos A. (2020). Role of Oncogenes and Tumor-suppressor Genes in Carcinogenesis: A Review. Anticancer Res..

[54] Huang D.W., Sherman B.T., Lempicki R.A. (2009). Systematic and integrative analysis of large gene lists using DAVID bioinformatics resources. Nat. Protoc..

[55] Grant T.J., Hua K., Singh A. (2016). Molecular Pathogenesis of Pancreatic Cancer. Prog. Mol. Biol. Transl. Sci..

[56] Reyes C.M., Dogruoz A., Istvanffy R., Friess H., Ceyhan G.O., Demir I.E. (2022). Molecular Profiling in Pancreatic Cancer: Current Role and Its Impact on Primary Surgery. Visc. Med..

[57] Collisson E.A., Bailey P., Chang D.K., Biankin A.V. (2019). Molecular subtypes of pancreatic cancer. Nat. Rev. Gastroenterol. Hepatol..

[58] Mizrahi J.D., Surana R., Valle J.W., Shroff R.T. (2020). Pancreatic cancer. Lancet.

[59] Kang Y., Kim Y.W., Kang J., Kim A. (2021). Histone H3K4me1 and H3K27ac play roles in nucleosome eviction and eRNA transcription, respectively, at enhancers. FASEB J. Off. Publ. Fed. Am. Soc. Exp. Biol..

[60] Khambata-Ford S., Liu Y., Gleason C., Dickson M., Altman R.B., Batzoglou S., Myers R.M. (2003). Identification of promoter regions in the human genome by using a retroviral plasmid library-based functional reporter gene assay. Genome Res..

[61] Dickel D.E., Zhu Y., Nord A.S., Wylie J.N., Akiyama J.A., Afzal V., Plajzer-Frick I., Kirkpatrick A., Gottgens B., Bruneau B.G. (2014). Function-based identification of mammalian enhancers using site-specific integration. Nat. Methods.

[62] Kashkin K.N., Chernov I.P., Stukacheva E.A., Kopantzev E.P., Monastyrskaya G.S., Uspenskaya N.Y., Sverdlov E.D. (2013). Cancer specificity of promoters of the genes involved in cell proliferation control. Acta Nat..

[63] Herz H.M. (2016). Enhancer deregulation in cancer and other diseases. BioEssays News Rev. Mol. Cell. Dev. Biol..

[64] Dietlein F., Wang A.B., Fagre C., Tang A., Besselink N.J.M., Cuppen E., Li C., Sunyaev S.R., Neal J.T., Van Allen E.M. (2022). Genome-wide analysis of somatic noncoding mutation patterns in cancer. Science.

[65] Zhang T., Zhang Z., Dong Q., Xiong J., Zhu B. (2020). Histone H3K27 acetylation is dispensable for enhancer activity in mouse embryonic stem cells. Genome Biol..

[66] Naldini L., Blomer U., Gage F.H., Trono D., Verma I.M. (1996). Efficient transfer, integration, and sustained long-term expression of the transgene in adult rat brains injected with a lentiviral vector. Proc. Natl. Acad. Sci. USA.

[67] Zufferey R., Nagy D., Mandel R.J., Naldini L., Trono D. (1997). Multiply attenuated lentiviral vector achieves efficient gene delivery in vivo. Nat. Biotechnol..

[68] Blankenberg D., Gordon A., Von Kuster G., Coraor N., Taylor J., Nekrutenko A., Galaxy T. (2010). Manipulation of FASTQ data with Galaxy. Bioinformatics.

[69] Langmead B., Salzberg S.L. (2012). Fast gapped-read alignment with Bowtie 2. Nat. Methods.

[70] Barnett D.W., Garrison E.K., Quinlan A.R., Stromberg M.P., Marth G.T. (2011). BamTools: A C++ API and toolkit for analyzing and managing BAM files. Bioinformatics.

[71] Quinlan A.R., Hall I.M. (2010). BEDTools: A flexible suite of utilities for comparing genomic features. Bioinformatics.

[72] Anders S., Pyl P.T., Huber W. (2015). HTSeq--a Python framework to work with high-throughput sequencing data. Bioinformatics.

[73] The ENCODE Project Consortium (2012). An integrated encyclopedia of DNA elements in the human genome. Nature.

[74] Wei Y., Zhang S., Shang S., Zhang B., Li S., Wang X., Wang F., Su J., Wu Q., Liu H. (2016). SEA: A super-enhancer archive. Nucleic Acids Res..

[75] Jiang Y., Qian F., Bai X., Liu Y., Wang Q., Ai B., Han X., Shi S., Zhang J., Li X. (2019). SEdb: A comprehensive human super-enhancer database. Nucleic Acids Res..

[76] Barrett T., Wilhite S.E., Ledoux P., Evangelista C., Kim I.F., Tomashevsky M., Marshall K.A., Phillippy K.H., Sherman P.M., Holko M. (2013). NCBI GEO: Archive for functional genomics data sets--update. Nucleic Acids Res..

[77] Afgan E., Baker D., van den Beek M., Blankenberg D., Bouvier D., Cech M., Chilton J., Clements D., Coraor N., Eberhard C. (2016). The Galaxy platform for accessible, reproducible and collaborative biomedical analyses: 2016 update. Nucleic Acids Res..

[78] Supek F., Bosnjak M., Skunca N., Smuc T. (2011). REVIGO summarizes and visualizes long lists of gene ontology terms. PLoS ONE.

