# Supplementary material for: Efficient Selection of Enhancers and Promoters from MIA PaCa-2 Pancreatic Cancer Cells by ChIP-lentiMPRA
Source: Int J Mol Sci. 2022 Nov 30;23(23):15011. doi: 10.3390/ijms232315011 (PMC9740945; doi:10.3390/ijms232315011)
Supplement: Supplementary file 1 [file ijms-23-15011-s001.zip › Supplementary Materials.pdf]

Supplementary Figures

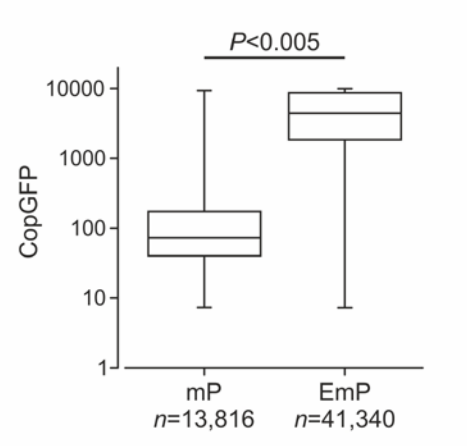

Figure S1. FACS analysis of MIA PaCa-2 cells transduced by basic (pLVPGm.1-mP) or control (pLVPGm.1-EmP) vectors. The cells were transduced using undiluted viral stocks and selected by puromycin. Ordinate axis (CopGFP) – fluorescence intensity. “Boxes and whiskers” show distribution of GFP-positive cells by fluorescence intensity in the populations of transduced cells (Mann-Whitney test, min-max range). *n* – number of GFP-positive cells.

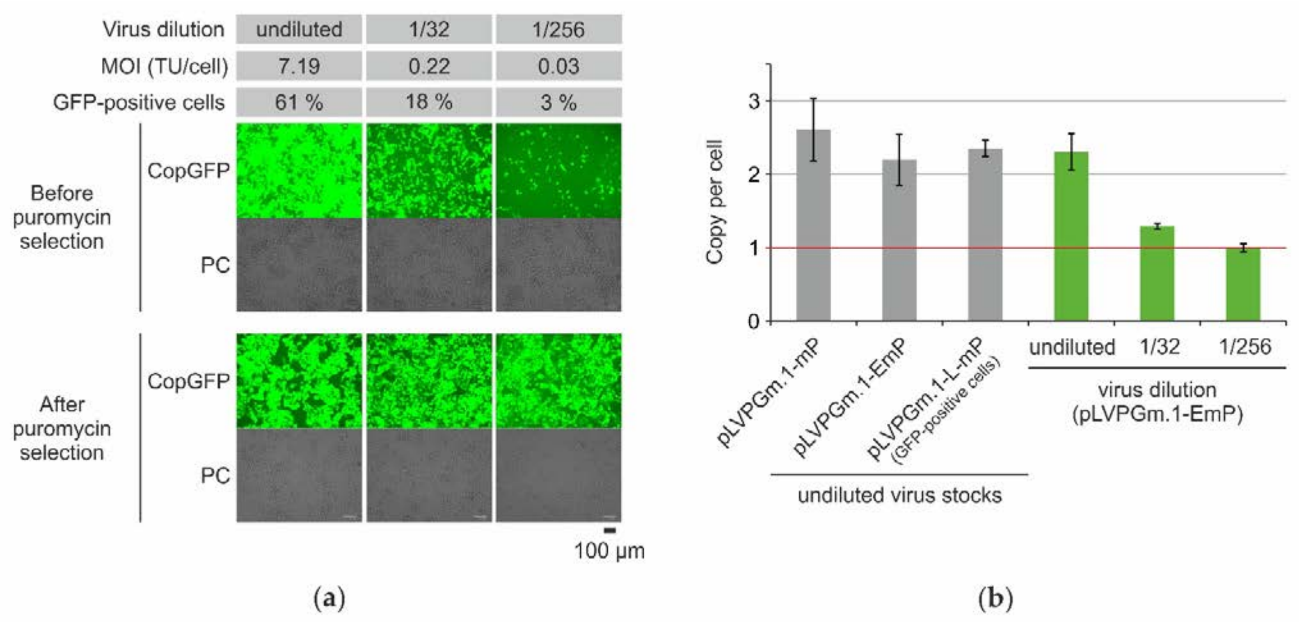

Figure S2. Determination of number of copies of integrated lentiviral DNA in populations of transduced MIA PaCa-2 cells after puromycin selection. (**a**) Production of MIAPaCa-2 cell populations transduced by control lentiviral vector pLVPGm.1-EmP with different multiplicity of infection (MOI). Cells were transduced by serial dilutions of control vector; virus titres were calculated by FACS-based titration method. The MOI value was less than 1 TU (transduced unit) per cell when using 1/32, 1/64 (not shown), 1/128 (not shown), and 1/256 dilutions of virus. CopGFP – fluorescent microscopy; PC – phase contrast microscopy. (**b**) Determination of lentiviral DNA copies in transduced cell populations by real-time qPCR. The number of copies of lentiviral DNA pLVPGm.1-EmP in the population of cells obtained using a dilution of the virus 1/256 (0.03 TU/cell) is taken as 1 copy per cell (red line). DNA samples received using undiluted viral stocks contain 2-3 copies of lentivirus per cell. pLVPGm.1-mP and pLVPGm.1-EmP – samples of genomic DNA isolated from puromycin resistant cells transduced with appropriate lentiviral vectors. pLVPGm.1-L-mP – genomic DNA isolated from GFP-positive cells transduced with pool of lentiviral vectors with fragments of ChIP DNA. All data were collected in triplicates and presented with standard deviations.

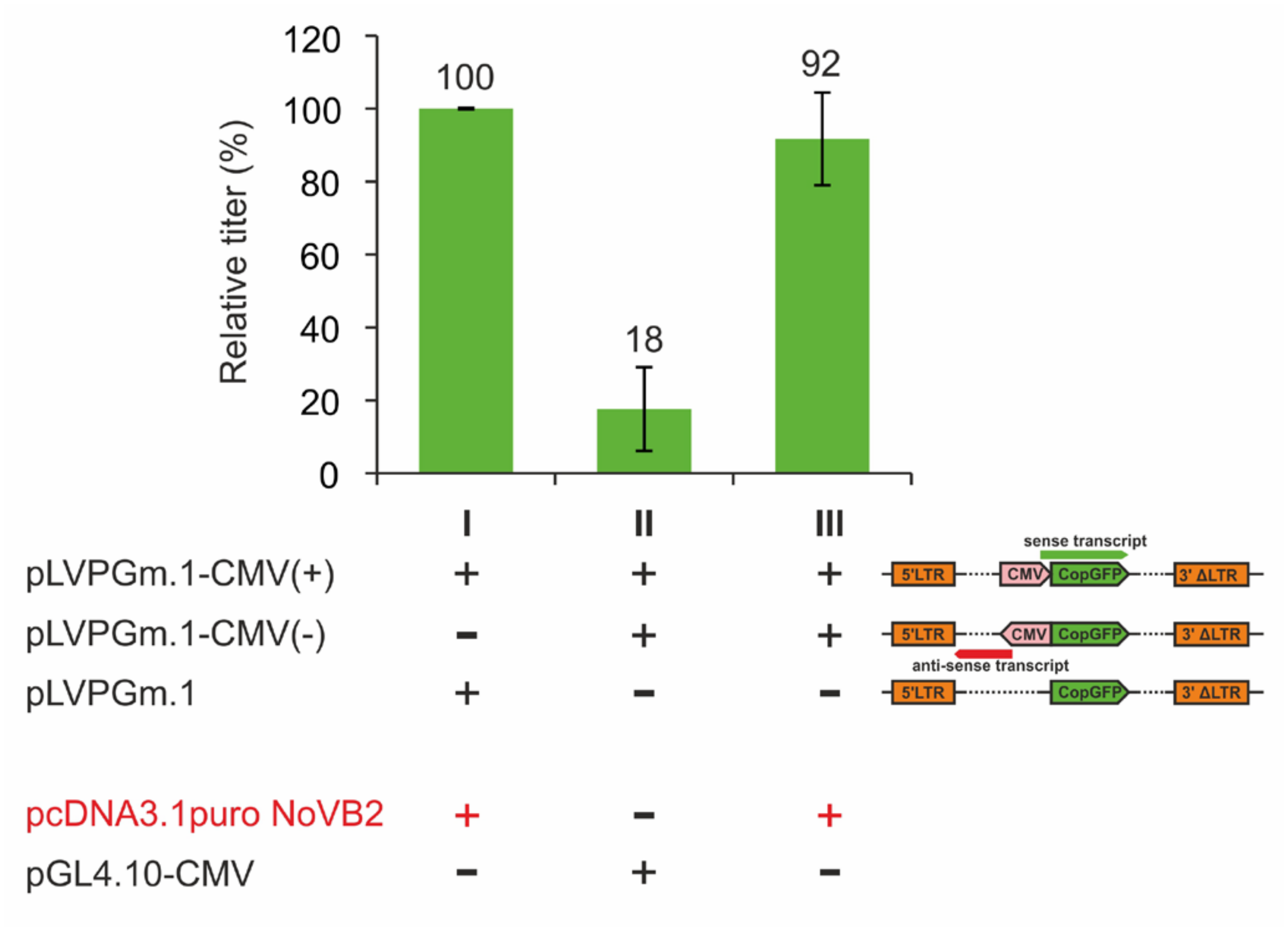

Figure S3. Inhibition of RNA-interference revokes decrease of lentivirus titre due to antisense transcription from lentiviral plasmid in transfected packing 293T cells. Titres were determined using FACS-based titration method on A431 cells. The mean of two measurements with standard deviations are presented. pLVPGm.1-CMV(+), lentiviral plasmid containing CMV promoter/enhancer in sense orientation. pLVPGm.1-CMV(–), lentiviral plasmid containing CMV promoter/enhancer in antisense orientation. pLVPGm.1, lentiviral plasmid without promoter in front of CopGFP gene. pcDNA3.1puro NoVB2, the plasmid encoding an interference inhibitor protein. pGL4.10-CMV, equilibration plasmid containing the luciferase gene under the control of the CMV promoter/enhancer.

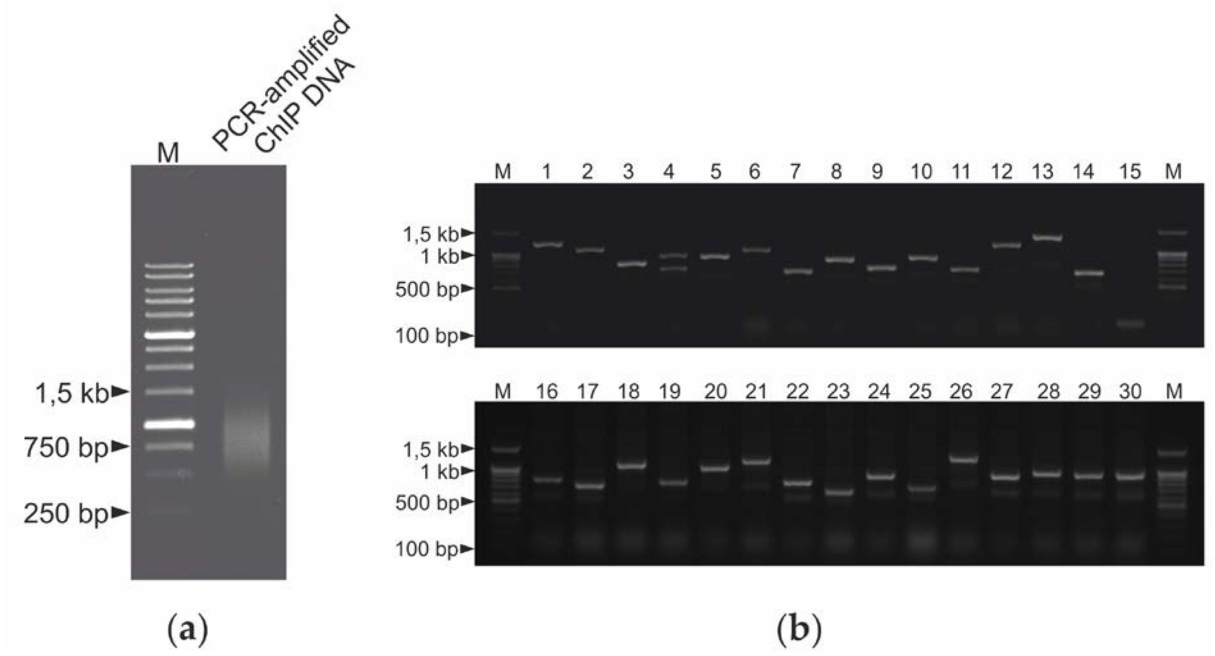

Figure S4. Preparation of the pLVPGm.1-L-mP lentiviral clone library. (**a**) Electrophoretic separation of PCR amplified fragments of ChIP DNA library in agarose gel. (**b**) PCR analysis of 30 random clones (nos. 1-30), from E.coli clonoteque transformed by lentiviral plasmids pLVPGm.1-L-mP with immunoprecipitated DNA fragments. M is a DNA marker.

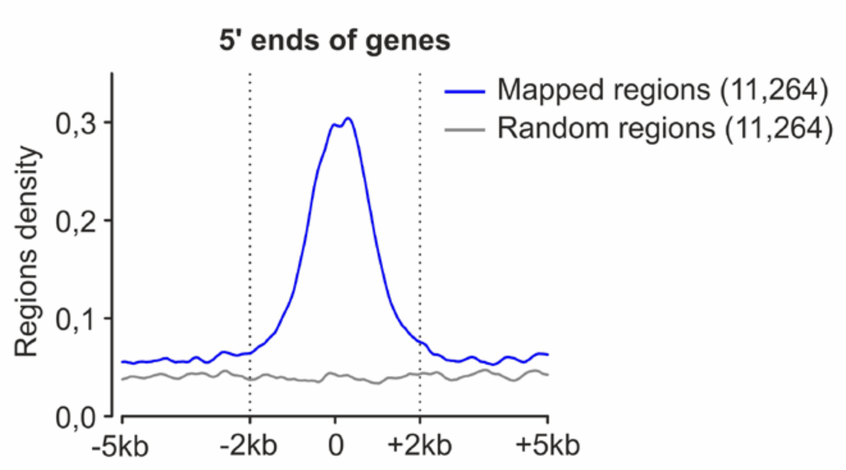

Figure S5. The mapped regions are concentrated near gene promoters. The results of the analysis of the distribution of mapped and random regions relative to the 5-ends of the genes are presented. The data were obtained using the SeqMonk software.

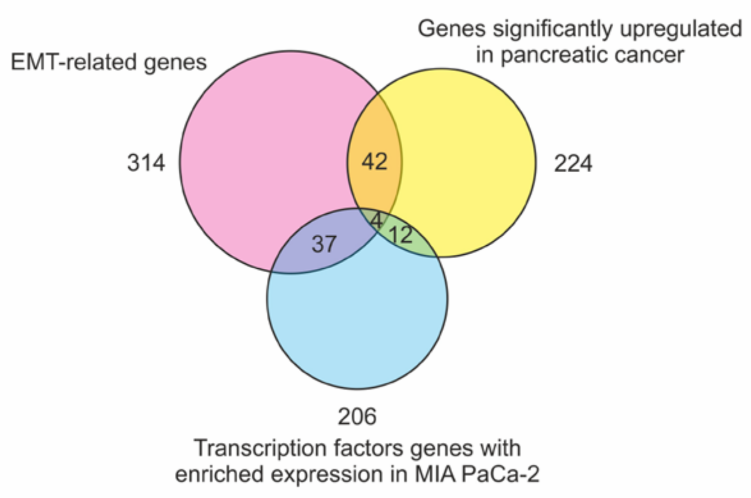

Figure S6. Representation of genes of the three functional categories among the genes associated with all the mapped regions. Data sources: dbEMT (Epithelial-mesenchymal transition gene database) – contains 1184 EMT-related genes (Zhao, Kong et al. 2015), the list of 827 genes significantly upregulated in pancreatic cancer (Goonesekere, Wang et al. 2014), and FANTOM5 SSTAR – contains 522 transcription factors genes with enriched expression in MIA PaCa2-2 cells (Abugessaisa, Shimoji et al. 2016).

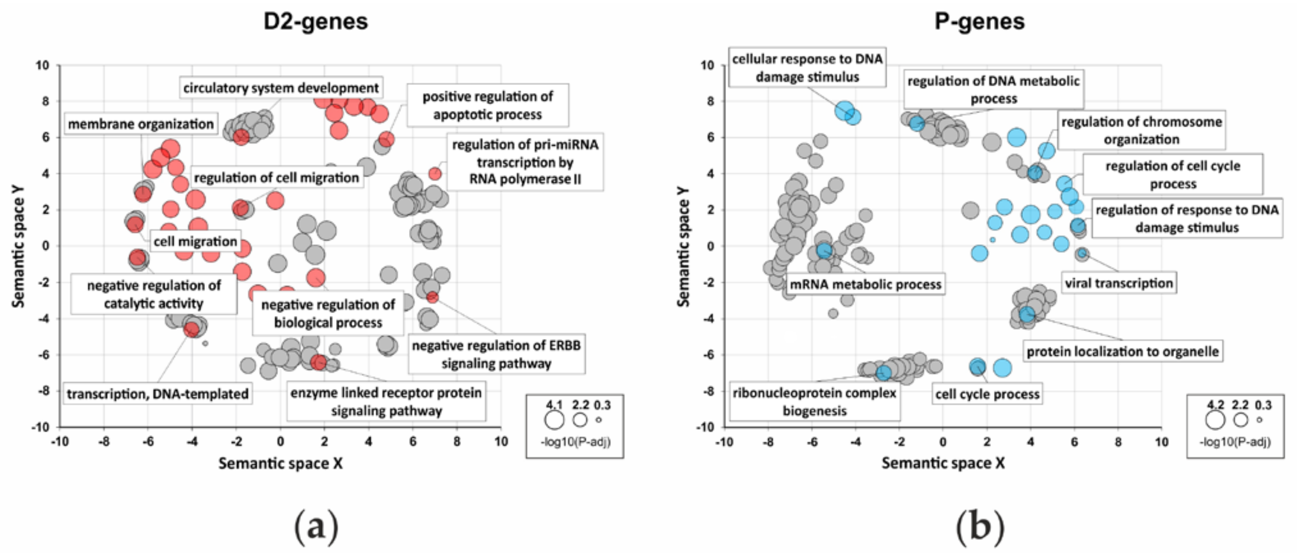

Figure S7. Functional annotation of genes associated with mapped regions. The 3798 genes associated with mapped regions were divided into three groups – D1, D2, and P (see the text). Each group were functionally annotated using DAVID (Huang da, Sherman et al. 2009), and REVIGO (Supek, Bošnjak et al. 2011) was used to visualize GO Biological Process Term clusters in semantic space. Circle sizes are proportional to -log10 p-values. (**a**) Functional annotation of genes associated with three or more distal regions (D2-genes). The most significant terms from each cluster are highlighted in red. (**b**) Functional annotation of genes associated with proximal regions (P-genes). The most significant terms from each cluster are highlighted in blue.

Supplementary Methods

4.1. ChIP DNA preparation and cloning

Approximately 1 × 10^7 logarithmically growing MIA PaCa-2 cells were fixed with 1 % (v/v) formaldehyde in DMEM/F-12 (1:1) medium (Invitrogen, 42400–010) at room temperature for 10 min. The fixed cells were washed twice with cold PBS containing 1 mM AEBSF and 2 µl/ml of a protease inhibitor cocktail (Sigma, P8340). Cells were pelleted at 700 g for 5 min at 4 °C and resuspended in 500 µl of lysis buffer (50 mM Tris-HCl pH 8, 1 % SDS, 10 mM EDTA). The lysate was incubated for 10 min on ice and sonicated with a Cole-Parmer CP750 ultrasonic processor (amplitude 30 %, pulse on 3 sec, pulse off 9.9 sec, total time 40 sec). The cell debris was removed using a microcentrifuge (10 min, 15 000 g, 4 °C). 20 µl aliquot was taken at this stage for subsequent use as the Input DNA control. The remaining supernatant was diluted 10-fold with IP buffer (16.7 mM Tris-HCl pH 8, 16.7 mM NaCl, 1.2 mM EDTA, 1 % Triton X-100, 0.01 % SDS, 1 mM AEBSF and 1 µl/ml of the protease inhibitor cocktail). The cell lysate was pre-cleared over 2 hours by incubation with 40 µl Dynabeads Protein G (Life Technologies) according to the manufacturer's recommendations. After removing beads, 2.5 mL of lysate was incubated with 4.5 µl of anti-H3K27ac antibodies (Abcam, ab4729) overnight at 4 °C with rotation. To collect immune complexes, 18 µl Dynabeads Protein G was added to the sample and incubated over 2 hours at 4 °C with rotation. Beads were washed successively with 900 µl of low salt wash buffer (20 mM Tris-HCl pH 8, 150 mM NaCl, 2mM EDTA, 1% Triton X-100, 0.1% SDS), 900 µl of high salt wash buffer (20 mM Tris-HCl pH 8, 500 mM NaCl, 2mM EDTA, 1% Triton X-100, 0.1% SDS), LiCl-containing wash buffer (10mM Tris-HCl pH 8, 1mM EDTA, 0.25M LiCl, 1% NP-40, 1% sodium deoxycholate) and twice with TE buffer (10mM Tris-HCl pH 8, 1mM EDTA). Immune complexes were eluted in 250 µl SDS elution buffer (1% SDS, 0.1M $NaHCO_3$) at 65 °C over 30 min with vortexing every 5 min. 20 µl of Input DNA sample was diluted with SDS elution buffer to 250 µl. Cross-links were reversed by addition of 10µL 5M NaCl to the samples and overnight heating at 65°C. 1 µl of 10 mg/mL RNase A was added to the samples and heating at 60 °C for 1 hour was performed, followed by addition of 10 µl 0.5 M EDTA, 20 µl 1M Tris-HCl pH 6.5 and 2 µl 10 mg/mL Proteinase K and heating at 56 °C for 1 hour. ChIP DNA and Input DNA were extracted using PCR purification kit (Qiagen, 28104). Aliquots were used to assess ChIP DNA enrichment by functional elements relative to Input DNA by quantitative real-time PCR (qPCR). Promoter and non-promoter regions from three loci of housekeeping genes *PSMB5*, *PSMB2* and *COPZ1* were amplified. Primers are listed in Table S5. Prior to amplification, an aliquot of Input DNA was diluted by 10 times. Each reaction contained 1 µl DNA template (ChIP DNA or diluted Input DNA), 5 µl 5X qPCRmix-HS SYBR (Evrogen, PK147S), 5 µl Primer mix (1 µM each), and 14 µl H_2O. qPCR was performed on LightCycler 480 II (Roche) with the following conditions: 95 °C, 5 min (hot start), 40 cycles of 95 °C, 30 sec; 63 °C, 20 sec; 72 °C, 30 sec. LinRegPCR program was used to analysis of qPCR data. The relative content of the analyzed regions in ChIP DNA was calculated as a percentage of Input DNA.

4.2. Lentivirus production

The day before the transfection, 293T cells were plated onto 75 cm^2 flask with density of $5x10^5$ cells/cm^2. The next day, cells were co-transfected by 12 µg lentiviral plasmid, 6 µg pMD.G [1], 12 µg pCMVΔR8.91 [2], and 7.5 µg pcDNA3.1 puro Nodamura B2 (Addgene, cat. #17228 [3]) using Lipofectamine 2000 (Invitrogen) according to the manufacturer's recommendations. If another flask were used, all components were scaled proportionally to the flask area. Briefly, on the day of the transfection, the medium in the flask was replaced with 12 ml Opti-MEM (Gibco). 75 µl Lipofectamine 2000 was diluted in 1.875 ml Opti-MEM and incubated for 5 min at room temperature. pDNAs were diluted in 1.875 ml Opti-MEM and combined with diluted Lipofectamine 2000. The solution was mix gently and incubated for 20 min at room temperature. After incubation, complexes were added to the flask containing 293T cells and Opti-MEM. Cells were incubated for 3.5 hours at 37 °C and 5 % CO_2 and the medium was replaced with 15 ml fresh DMEM/F-12 (1:1) medium (Invitrogen, 42400–010) supplemented with 10 % fetal calf serum. Transfected cells were incubated for 24 hours at 37 °C, then placed at 32 °C and incubated for another 24 hours. The virus-containing medium was centrifuged at 700 g for 5 min at 4 °C, and the supernatant was filtered using 0.45 µm PVDF filter. Purified viral-containing mediums (virus stocks) were immediately used for MIA PaCa-2 cells transduction.

4.3. FACS-based titration of control lentiviral vector pLVPGm.1-EmP.

MIA PaCa-2 cells were seeded in 1 ml of DMEM/F-12 medium (1:1) supplemented with 10 % fetal calf serum (FCS) per well of a 24-well plate. Cells were incubated at 37 °C and 5 % CO_2 overnight. The next day, the average number of cells per well was 96×10^3. The fresh virus stock obtained by using control lentiviral vector pLVPGm.1-EmP was diluted with fresh medium to obtain various dilutions (1/32, 1/64, 1/128, 1/256). The MIA PaCa-2 cells were transduced with both non-diluted virus and with prepared dilutions. For this, the old medium in the wells was removed and 300 µl of each virus-containing medium was added per well in duplicates. 300 µl of fresh medium was added in each well. Cells were incubated at 37 °C and 5 % CO_2 overnight. The viral-containing medium was replaced with 1 ml fresh DMEM/F-12 (1:1) medium (Invitrogen, 42400–010) supplemented with 10 % fetal calf serum per well and cells were incubated for 72 hours at 37 °C and 5 % CO_2. Then the numbers of GFP-positive cells in each well were determined by flow cytometry. The titer was calculated using a percentage of GFP-positive cells within the range 1-15 % using the following formula: titer = (F x C x D)/V, where F – frequency of GFP-positive cells, C – average number of cells per well on the day of transduction, D – coefficient of lentivirus dilution, and V – Volume of virus added per well. The virus titer was 2.3×10^6 TU/ml.

4.4. Adapter ligation and PCR.

The End Repair/dA-Tailing Module (NEB, E7442S) was used to convert ChIP DNA to repaired DNA having 5' phosphorylated dA-tailed ends. The DNA was then cleaned up using 1.6x volume of Agencourt AMPure XP beads (Beckman Coulter, A63880) according to the manufacturer's recommendations and eluted in 15 µl H_2O. Repaired DNA fragments were ligated to adapter, obtained by annealing of equimolar amounts of the two oligonucleotides (oligo 1 and oligo 2, Table S5). The ligation mixture contained 15 µl of repaired ChIP DNA, 2.5 µl of 10 µM adapter, 2.5 µl of 10x T4 DNA ligase buffer, and 5 µl of 30 Weiss U/µl T4 DNA ligase (Thermo Scientific, EL0013) was incubated at 16 °C overnight. After the ligation, the DNA was purified using Agencourt AMPure XP beads and eluted in 10 µl H_2O. 10 µl of eluted DNA was incubated with preheated (98 °C, 45 sec) mixture contained 25 µl KAPA HiFi HotStart ReadyMix (2X) (Kapa Biosystems, KM2605) and 13 µl of H_2O at 72 °C over 90 sec for blunting ends by filling-in 5'-overhangs. Then 2 µl of 10 µM Library Primer (Table S5) was added to the mixture and 10 cycles of pre-amplification were performed (98 °C, 15 sec; 61 °C, 30 sec; 72 °C, 30 sec). Pre-amplified ChIP DNA was subjected to preparative PCR amplification in 5 reactions (2 µl of pre-amplified ChIP DNA, 1 µl of 10 µM Library Primer, 12.5 µl of KAPA HiFi HotStart ReadyMix (2X), and 9.5 µl of H_2O per reaction) with the following conditions: 10 cycles of 98 °C, 15 sec; 61 °C, 30 sec; 72 °C, 30 sec, and final extension at 72 °C for 1 min. PCR-amplified ChIP DNA was purified by phenol-chloroform extraction and ethanol precipitation. An aliquot was used

to assess amplified library enrichment by functional elements relative to Input DNA by quantitative real-time PCR (qPCR, see ChIP DNA preparation and cloning).

4.5. ChIP DNA cloning.

PCR-amplified ChIP DNA was digested with Xho I restriction endonuclease over 4 hours, purified with PCR purification kit (Qiagen, #28104) and inserted into XhoI- and SalI-treated pLVPGm.1-mP using standard ligase-dependent molecular cloning procedures. After the ligation, DNA was purified by phenol-chloroform extraction, precipitated with ethanol and dissolved in 10 µl of H_2O.

Electro-competent DH5-alpha *E. coli* were prepared as described in the instruction manual "Gene Pulser Xcell Electroporation System" (Bio-Rad). 2 µl of ligated DNA were used for electroporation of 40 µl electro-competent *E.coli* per reaction. In sum, 5 reactions were used. Electroporation was performed in 0.2 cm cuvette by Gene Pulser Xcell system (Bio-Rad, 165-2660) using the manufacturer's pre-set protocol "Bacterial 2". After the electroporation, E.coli were transferred into 200 ml SOB medium and grown for 50 min with rotation. To assess cloning efficiency, 1/20000 part was seeded on an agar plate containing 100 µg/ml of ampicillin. Ampicillin was added to the SOB medium with *E.coli* to final concentration of 100 µg/ml and cells were grown overnight with rotation. Approximately $20x10^6$ of transformants were obtained. Cells were pelleted by centrifugation. A pool of plasmid DNAs (pLVPGm.1-mP-L) with ChIP DNA library fragments was isolated from precipitated cells using Midipreps DNA Purification System (Promega, A7640). An aliquot was used to assess cloned ChIP DNA enrichment by functional elements relative to Input DNA by quantitative real-time PCR (qPCR, see ChIP DNA preparation and cloning).

30 colonies grown on an agar plate were analyzed by PCR with primers P1 and P2 flanking inserted DNA fragments (Table S5). Each colony was transferred into 5 µl of H_2O and heated at 95 °C for 5 min. Then 15 µl of mixture containing 4 µl of ScreenMix (2X) (Evrogen, PK041S), 1 µl of Primer mix (P1 and P2, 5 µM each), and 10 µl of H_2O was added to heated bacteria and amplified with following conditions: 24 cycles of 95 °C, 20 sec; 61 °C, 20 sec; 72 °C, 75 sec. PCR products were analyzed using agarose gel electrophoresis. All colonies (100 %) contained inserts with average size of ~700 bp.

4.6. Massive functional selection of enhancers and promoters, active in MIA PaCa-2 cells.

4.6.1. Transduction of MIA PaCa-2 cells

The day before transduction, MIA PaCa-2 cells were plated onto 75 cm^2 flask with density $4x10^4$ cells/cm^2. Cells were incubated at 37 °C and 5% CO_2 overnight. The medium in the flask was replaced with 15 ml fresh virus stock obtained by using pool of lentiviral plasmids with ChIP DNA fragments (pLVPGm.1-L-mP) with 3 ml fresh DMEM/F-12 (1:1) medium supplemented with 10 % fetal calf serum. Cells were incubated at 37 °C and 5% CO_2 overnight. The viral-containing medium was replaced with 15 ml fresh medium supplemented with fetal calf serum. One day later, the medium was replaced with 15 ml fresh medium containing puromycin (3 µg/ml). Over 48 hours, the medium was replaced with fresh puromycin containing medium. The cells were incubated for 24 hours and plated onto two 75 cm^2 flasks in puromicin containing medium. After 24 hours of incubation, GFP-positive cells with higher fluorescence signal were selected using BD FACSAria III cell sorter. Approximately 37 x 10^3 of transduced GFP-positive cells were collected by three FACS-selections. After each FACS, cells were cultured followed by extraction of gDNA using QIAamp DNA Mini kit (Promega, cat. #51304).

4.6.2. PCR amplification of inserts and functional assay.

Genomic DNA (gDNA) was isolated from GFP-positive cells and amplified by nested-PCR using KAPA HiFi HotStart DNA Polymerase (Kapa Biosystems). In the first step, 25 µl PCR amplification was performed using 260 ng of gDNA and primers P3 and P4 (Table S5) under the following conditions: initial activation at 98 °C for 45 sec,

12 cycles of 98 °C 20 sec/61 °C 20 sec/72 °C 70 sec, and final extension at 72 °C for 90 sec. After amplification, DNA was purified using a PCR purification kit (Qiagen, 28104) and eluted in a final volume of 50 µl. In the second nested-PCR step, amplification was performed in 5 reactions of 25 µl each using purified DNA (10 µl per reaction) and primers P1 and P2 (0.4 pmol of each per reaction) (Table X) under the following conditions: initial activation at 98 °C for 45 sec, 14 cycles of 98 °C 20 sec/62 °C 20 sec/72 °C 50 sec and final extension at 72 °C for 60 sec. The amplified DNA was purified using the PCR purification kit (Qiagen, 28104).

The isolated DNA was sequentially treated with XhoI and SbfI restrictases, ligated into the pGRm.1-mP plasmid cut at the XhoI and SbfI sites and chemical transformation of E. coli DH-5alpha was performed. Night cultures were obtained from 28 E.coli clones containing inserts. Plasmid DNAs (pDNAs) were isolated using Plasmid Miniprep (Evrogen, BC021) and transfected into MIA PaCa-2 cells. The day before transfection, MIA PaCa-2 cells were seeded into 24-well plates at a density of $8x10^5$ cells/cm2. Transfection was performed with a 100 µl mixture (per well) containing 800 ng pDNA, 2 µl Lipofectamin 2000 (Invitrogen, 11668-019) and Opti-MEM (Gibco, 31985070), according to Invitrogen recommendations. After 3.5 hours of transfection, the medium was replaced with DMEM/F-12 (1:1) medium (Invitrogen, 42400-010) supplemented with 10% fetal calf serum and incubated for 48 hours. After transfection, cell populations were analyzed using a ZOE Fluorescent Cell Imager (Bio-Rad) and a DB FACScan flow cytofluorimeter (Becton Dickinson).

4.6.3. Libraries preparation and next-generation sequencing

gDNA samples isolated from GFP-positive cell populations were amplified by three sequential PCR using KAPA HiFi HotStart DNA Polymerase (2X) (Kapa Biosystems). Illumina recommendations based on the "16S Metagenomic Sequencing Library Preparation" protocol were used for primer design and PCR. The first PCR was performed using external primers P3 and P4 (Table X) flanking the lentivirus cloned fragments. Each sample was amplified using 5 reactions. Each 25 µL reaction contained 200-250 ng of gDNA, 2 µL of primer mixture (5 µM each), and polymerase. PCR was performed under the following conditions: initial activation at 95 °C for 3 min, 12 cycles of 98 °C 20 sec/65 °C 20 sec/72 °C 70 sec, and final extension at 72 °C for 60 sec. DNA was purified using Agencourt AMPure XP beads (Beckman Coulter, A63880) according to the manufacturer's recommendations using 1:1 ratio. The second PCR was performed using internal primers, R1M15f and R2M15r (Table X), flanking fragments were cloned into lentivirus and containing sections of Illumina adaptor sequences required for paired-end sequencing. PCR was performed in a 50 µl mixture containing 21 µl of purified PCR product, 4 µl of primer mixture (2.5 µM each), and polymerase under the following conditions: initial activation at 95 °C for 3 min, 8 cycles of 98 °C, 20 sec; 62 °C, 20 sec; 72 °C, 60 sec, and final extension at 72 °C for 60 sec. DNA was purified using Agencourt AMPure XP beads (Beckman Coulter, A63880) according to the manufacturer's recommendations using 1:1 ratio. The third PCR was performed using standard Illumina primers, P5S502 and P7N70X (Table S5), containing additional adaptor sequences required for paired-end sequencing. The P7N70X primer contained an 8 bp Index sequence unique to each resulting library. For each sample, one 25 µl reaction was performed with 10.5 µl of purified PCR product, 2 µl of primer mixture (2.5 µM each), and polymerase, under the following conditions: initial activation at 95 °C for 3 min, 8 cycles of 98 °C, 30 sec; 55 °C, 30 sec; 72 °C, 60 sec, and final extension at 72 °C for 5 min. Libraries of DNA fragments ranging 400 to 1400 bp were purified using agarose gel electrophoresis, mixed and sequenced on Illumina MiSeq at both ends (2 × 150 bp).

References

1. Naldini, L.; Blomer, U.; Gage, F.H.; Trono, D.; Verma, I.M. Efficient transfer, integration, and sustained long-term expression of the transgene in adult rat brains injected with a lentiviral vector. *Proc Natl Acad Sci U S A* **1996**, *93*, 11382-11388. https://doi.org/10.1073/pnas.93.21.11382.
2. Zufferey, R.; Nagy, D.; Mandel, R.J.; Naldini, L.; Trono, D. Multiply attenuated lentiviral vector achieves efficient gene delivery in vivo. *Nature biotechnology* **1997**, *15*, 871-875. https://doi.org/10.1038/nbt0997-871.
3. Sullivan, C.S.; Ganem, D. A virus-encoded inhibitor that blocks rna interference in mammalian cells. *J Virol* **2005**, *79*, 7371-7379. https://doi.org/10.1128/JVI.79.12.7371-7379.2005.
