# Supplementary material for: Efficient Selection of Enhancers and Promoters from MIA PaCa-2 Pancreatic Cancer Cells by ChIP-lentiMPRA
Source: Int J Mol Sci. 2022 Nov 30;23(23):15011. doi: 10.3390/ijms232315011 (PMC9740945; doi:10.3390/ijms232315011)
Supplement: Supplementary file 1 [file ijms-23-15011-s001.zip › Table_S1.pdf]

Table S1. Proportion of mapped fragments of the studied DNA libraries that overlap with various functional sites in the genome

Dataset (N)	Dnase I[1]	TFBS[2]	SEA SE[3]	H3K27ac[4]	CAGE[5]	SE MIA PaCa-2[6]
L1 (6244)	75,3	77,9	49,7	48,4	20,3	8,8
L2 (5245)	74,6	76,9	49,8	48,4	18,6	8,9
Mapped Regions (11264)	74,7	77,2	49,6	47,7	19,1	8,7
Discarded Regions (11171)	62,8	65,7	41,5	31,9	13,0	5,9
Random Regions (11264)	42,1	46,0	27,5	5,7	2,7	0,8

max

min

[1] DNase I Hypersensitivity Peak Clusters from ENCODE (95 cell types)

[2] Transcription Factor ChIP-seq Clusters (340 factors, 129 cell types) from ENCODE 3

[3] Super-enhancers from SEA database (http://sea.edbc.org/)

[4] H3K27ac ChIP-seq peaks in MIA PaCa-2 cell line (Diaferia et al., 2016)

[5] CAGE peaks from FANTOM5 project (https://fantom.gsc.riken.jp/5/)

[6] MIA PaCa-2 super-enhancers from SEdb 2.0 (http://www.licpathway.net/sedb)
