# Supplementary material for: Efficient Selection of Enhancers and Promoters from MIA PaCa-2 Pancreatic Cancer Cells by ChIP-lentiMPRA
Source: Int J Mol Sci. 2022 Nov 30;23(23):15011. doi: 10.3390/ijms232315011 (PMC9740945; doi:10.3390/ijms232315011)
Supplement: Supplementary file 1 [file ijms-23-15011-s001.zip › Table_S3.pdf]

Table S3. Proportion of mapped regions overlapping with different functional regions in the genome.

Regions	Dnase I[1] (%)	TFBS[2] (%)	SEA SE[3] (%)	CAGE[4] (%)
Mapped Regions (MR)	74.7	77.2	49.6	19.1
Proximal MR	97.9	99.0	37.8	55.5
Distal MR	68.7	71.9	54.8	8.0
H3K27ac+ MR	93.3	94.6	59.3	36.3
Proximal MR	98.9	99.6	38.4	57.3
Distal MR	88.8	90.6	78.7	17.5
H3K27ac- MR	57.7	61.3	40.7	3.5
Random Regions	42.1	46.0	27.5	2.7

max

min

[1] DNase I Hypersensitivity Peak Clusters from ENCODE (95 cell types)

[2] Transcription Factor ChIP-seq Clusters (340 factors, 129 cell types) from ENCODE 3

[3] Super-enhancers from SEA database (http://sea.edbc.org/)

[4] CAGE peaks from FANTOM5 project (https://fantom.gsc.riken.jp/5/)
