# Supplementary material for: Efficient Selection of Enhancers and Promoters from MIA PaCa-2 Pancreatic Cancer Cells by ChIP-lentiMPRA
Source: Int J Mol Sci. 2022 Nov 30;23(23):15011. doi: 10.3390/ijms232315011 (PMC9740945; doi:10.3390/ijms232315011)
Supplement: Supplementary file 1 [file ijms-23-15011-s001.zip › Table_S5.pdf]

Table S5. Oligonucleotides used in the work

Name	Sequence (5'-3')	Description
PSMB5pr for<br>PSMB5pr rev	CGATTCCTGGCTCTTCTGGG<br>AGGTCCAGGGAGTCTCAGTG	PSMB5 promoter region
PSMB5g for<br>PSMB5g rev	ACATACCACCCCATCTCACC<br>GCTTGAAACCTAAGTCATTTGGA	Non-promoter intragenic region of PSMB5
PSMB2pr for<br>PSMB2pr rev	ACCTCAGAGCGAAGATTGGC<br>ACTGAACATAAGGCACCCGG	PSMB2 promoter region
PSMB2o for<br>PSMB2o rev	TGTGTCCAGAGTGCCTTTCA<br>TCAGGGCAGCAACTGTGTAG	Non-promoter intergenic region of PSMB5 locus
PSMB2g2 for<br>PSMB2g2 rev	TGCCAGACCACCACTACAAA<br>GGGAAAACCTCCCTAGCAAA	Non-promoter intragenic region of PSMB2
COPZ1pr for<br>COPZ1pr rev	GAACGTATGGCCAATCAGCG<br>ACCAAAATCAGCGCCTCCAT	COPZ1 promoter region
COPZ1g for<br>COPZ1g rev	CCGTCAGTTCTTCCCGAGT<br>AGATTCAAAAAGCACAGCTCA	Non-promoter intragenic region of COPZ1
oligo 1<br>oligo 2	CTCGAGGACTTC<br>GCTGAGGTCGACTATCGTAGAAGTCCTCGAGT	Adapter oligos. Adapter contains library primer annealing region and XhoI restriction site for cloning ChIP DNA
Library Primer	GCTGAGGTCGACTATCGTAGAAGT	Library Primer are used for PCR amplification ChIP DNA fragments with adapters on the ends
P1<br>P2	CAGGAAACACGTATGAC<br>GTAAAACGACGGCCAG	Inner Forward primer (nested PCR)<br>Inner Reverse primer (nested PCR)
P3<br>P4	GTAAAACGACGGCCAG<br>GTAAAACGACGGCCAG	Outer Forward primer (nested PCR)<br>Outer Reverse primer (nested PCR)
R1M15f	TCGTCGGCAGCGTCAGATGTGTATAAGAGACA GGTAAAACGACGGCCAGGC	PCR rescue
R2M15r	GTCTCGTGGGCTCGGAGATGTGTATAAGAGAC AGCAGGAAACACGTATGACCT	PCR rescue
P5S502	AATGATACGGCGACCACCGAGATCTACACCTC TCTATTCGTCGGCAGCGTC	PCR rescue
P7N70X	CAAGCAGAAGACGGCATACGAGAT[i7]GTCTCG TGGGCTCGG	PCR rescue
